# Mass cytometry analysis reveals a distinct immune environment in peritoneal fluid in endometriosis: a characterisation study

**DOI:** 10.1186/s12916-019-1470-y

**Published:** 2020-01-07

**Authors:** Manman Guo, Cemsel Bafligil, Thomas Tapmeier, Carol Hubbard, Sanjiv Manek, Catherine Shang, Fernando O. Martinez, Nicole Schmidt, Maik Obendorf, Holger Hess-Stumpp, Thomas M. Zollner, Stephen Kennedy, Christian M. Becker, Krina T. Zondervan, Adam P. Cribbs, Udo Oppermann

**Affiliations:** 1 Botnar Research Centre, NIHR Biomedical Research Unit Oxford, Nuffield Department of Musculoskeletal Sciences, University of Oxford, Oxford, UK; 2 Nuffield Department of Women’s and Reproductive Health, University of Oxford, Oxford, UK; 3 Bayer AG, Drug Discovery Pharmaceuticals, Gynecological Therapies, Müllerstr. 178, Berlin, Germany; 4 The Wellcome Trust Centre for Human Genetics, University of Oxford, Oxford, UK; 5 Freiburg Institute for Advanced Studies (FRIAS), University of Freiburg, Freiburg im Breisgau, Germany

**Keywords:** Endometriosis, Mass cytometry, Peritoneal fluid, Peripheral blood, Immune cells, Innate immunity, Adaptive immunity, CD69

## Abstract

**Background:**

Endometriosis is a gynaecological condition characterised by immune cell infiltration and distinct inflammatory signatures found in the peritoneal cavity. In this study, we aim to characterise the immune microenvironment in samples isolated from the peritoneal cavity in patients with endometriosis.

**Methods:**

We applied mass cytometry (CyTOF), a recently developed multiparameter single-cell technique, in order to characterise and quantify the immune cells found in peritoneal fluid and peripheral blood from endometriosis and control patients.

**Results:**

Our results demonstrate the presence of more than 40 different distinct immune cell types within the peritoneal cavity. This suggests that there is a complex and highly heterogeneous inflammatory microenvironment underpinning the pathology of endometriosis. Stratification by clinical disease stages reveals a dynamic spectrum of cell signatures suggesting that adaptations in the inflammatory system occur due to the severity of the disease. Notably, among the inflammatory microenvironment in peritoneal fluid (PF), the presence of CD69^+^ T cell subsets is increased in endometriosis when compared to control patient samples. On these CD69^+^ cells, the expression of markers associated with T cell function are reduced in PF samples compared to blood. Comparisons between CD69^+^ and CD69^−^ populations reveal distinct phenotypes across peritoneal T cell lineages. Taken together, our results suggest that both the innate and the adaptive immune system play roles in endometriosis.

**Conclusions:**

This study provides a systematic characterisation of the specific immune environment in the peritoneal cavity and identifies cell immune signatures associated with endometriosis. Overall, our results provide novel insights into the specific cell phenotypes governing inflammation in patients with endometriosis. This prospective study offers a useful resource for understanding disease pathology and opportunities for identifying therapeutic targets.

## Background

Endometriosis is a gynaecological disease characterised by the growth of endometrial-like tissues in ectopic, often peritoneal locations where they develop and bleed in response to the hormones of the menstrual cycle, frequently leading to chronic pelvic pain and subfertility. It affects millions of women worldwide with an estimated population prevalence of 6–10% of women of reproductive age and up to 25–50% in women seeking treatment for infertility [1]. Retrograde menstruation through the fallopian tubes into the pelvis followed by attachment of endometrial tissue at ectopic locations within the peritoneal cavity during menstrual flow is the most widely accepted causal factor [2]. However, not all women with retrograde menstruation develop endometriosis, suggesting that there are additional factors involved in the survival of mislocated cells and their development into endometriotic lesions, such as genetic susceptibility, autoimmunity, or anomalous inflammatory responses [3–6]. Immune dysfunctions have been suggested to contribute to the development and progression of endometriosis by creating a microenvironment that supports the survival and implantation of endometriotic cells [7–9]. Indeed, development of endometriosis is associated with sustained peritoneal inflammation, including increases in peritoneal fluid (PF) volume and white blood cell concentrations in the peritoneal cavity [8, 10]. Arising mainly from ovarian exudation, PF contains a variety of immune cells and secreted products (e.g. cytokines, growth factors, steroid hormones), creating an inflammatory microenvironment that can assist the growth and maintenance of endometriotic lesions [11, 12]. As the front line of innate immunity, macrophages have been identified as the largest immune population in the PF of endometriosis patients, and they were found to be alternatively activated [13, 14] based on the M1/M2 paradigm which has recently been extended and refined [15, 16]. In contrast to classically activated (M1) macrophages that display a pro-inflammatory phenotype with IFN-γ as its signature cytokine, alternatively activated (M2) macrophages characterised by IL-4/IL-13 signatures are considered to resolve inflammatory responses and promote angiogenesis and tissue repair [17, 18], thereby possibly facilitating growth of endometriotic lesions. This view is supported by experiments where injection of M2 macrophages in murine endometriosis models enhances the growth of ectopic lesions, in contrast to M1 macrophages, which were protective from endometriosis [10, 13]. However, the simplified view of M1/M2 macrophage biology has now been extensively modified, suggesting a greater degree of plasticity of macrophage responses to different stimuli in the microenvironment [19, 20].

In addition to macrophages, other innate immune cells have been proposed as important elements in endometriosis pathogenesis. The establishment of endometriotic lesions suggests a possible defect in lesion clearance by natural killer (NK) cells within the peritoneal cavity. Decreased NK cell cytotoxicity in endometriosis patients has been reported [21, 22], although D’Hooghe et al. detected no change in lymphocyte-mediated cytotoxicity and NK cell activity in baboons with endometriosis [23]. Although the adaptive immunity in endometriosis is less defined, an aberrant T cell response also appears to be a signature of endometriosis since an increased proportion of immunosuppressive regulatory T (Treg) cells in the PF of women with endometriosis has been reported [24, 25]. Treg cells may play a role in endometriosis by controlling an effector cell network including macrophages, NK cells, dendritic cells, and cytotoxic T cells, where an increase of immunosuppressive Treg cell activity is possibly associated with the observed lack of tissue clearance in endometriosis [26, 27].

Previous studies have relied on well-established, fluorescence detection-based flow cytometry techniques, whereas only recently, high-resolution single-cell techniques have become available that permit a more detailed analysis of immune cell populations. Mass cytometry, also named as *Cytometry by Time-Of-Flight* (CyTOF), is a recently developed technique that enables multiparametric single-cell analysis. Using stable metal isotopes as reporters, this approach overcomes many limitations of traditional flow cytometry and currently detects up to 40 parameters in a single sample [28], making it particularly powerful in studies with patient samples [29, 30]. The goal of this study was to identify clinically relevant immune cell subtypes implicated in endometriosis. Using a panel of antibodies to label major haematopoietic cell types, we present a single-cell investigation in which we characterise the peritoneal immune cell composition in patients with and without endometriosis. The study offers a systematic view of immune cell signatures found in the peritoneal cavity and reveals CD69^+^ T cell populations that are associated with endometriosis.

## Methods

### Sample collection

Matched peritoneal fluid and peripheral blood samples from consented endometriosis patients and non-endometriosis controls were collected as part of the ENDOX study from patients undergoing laparoscopic surgery at the Women’s Centre, John Radcliffe Hospital, Oxford, UK (REC reference 09/H0604/58). Venous blood samples were drawn from patients in the morning on the day of surgery. Peritoneal fluid was collected during laparoscopic surgery before any surgical procedure was performed to avoid contamination from blood. Paired samples, where peritoneal fluid was contaminated by blood, were not used in the study. Surgery, sample collection, and processing were performed locally within the Oxford Hospital area. Tissue was collected according to standard operating procedures to maintain the highest quality, while minimising the time to processing. In order to achieve the highest reproducibility and consistency in sample collection, sample processing times ranged from 2 to 4 h (samples were all collected within 1–2 h of laparoscopy); any sample falling outside this collection window were discarded and not used within this study. Patient demographics for this study are listed in Additional file 1: Tables S2, S5, and S7.

### Preparation of cells from PF and blood samples

Cells were pelleted from PF and washed three times in cold PBS by centrifugation at 1800 rpm for 5 min. PF cells were counted and stored in FBS/10% DMSO at − 80 °C until analysis. Red blood cells from blood samples were lysed using RBC lysis buffer (BioLegend) according to the manufacturer’s instructions, then washed in cold PBS by centrifugation at 1800 rpm for 5 min. Blood cells were counted and frozen in FBS/10% DMSO at − 80 °C for later analysis. Cell viability was verified by counting live and dead cells before and after freezing process using Trypan blue (Sigma-Aldrich), and cell viability of samples used for the study was above 70% for PF cells and 90% for blood cells.

### Antibodies

Pre-conjugated antibodies were purchased from DVS Sciences, and purified antibodies were purchased from BioLegend, R&D Systems, or Abcam (listed in Additional file 1: Tables S1, S2, S3, S4, S5, S6, and S7). Purified antibodies were labelled with corresponding metal tags using Maxpar® Antibody Labeling Kits (DVS Sciences) as per manufacturer’s instructions and titrated to determine the working concentration.

### CyTOF staining and barcoding

All buffers and reagents used in this section were purchased from Fluidigm, unless otherwise stated. Cryopreserved cells were removed from the freezer and immediately thawed at 37 °C in a water bath. Cells were then washed with complete RPMI (Sigma) followed by three washes in Barium-free PBS (Sigma) by spinning at 1800 rpm for 3 min. From 200,000 to one million cells from each sample were stained with intercalator-103Rh to a final concentration of 25 μM for 20 min at room temperature (RT) to label dead cells. After one wash in MaxPar staining buffer, cells were fixed in Fix I Buffer for 10 min at RT followed by two washes in barcode perm buffer for permeabilisation. Each sample was labelled with barcodes from Cell-ID™ 20-plex Pd barcoding kit in 100 μl barcode perm buffer respectively by incubating for 30 min. Barcoded samples were washed twice in MaxPar staining buffer and pooled into one sample. Human TruStain FcX Fc receptor blocker (BioLegend) was used to block Fc receptors of cells, which were then incubated with cell surface antibodies as listed in Additional file 1: Table S1, S4, or S6 at 4 °C for 30 min. After incubation, cells were washed twice in MaxPar staining buffer and fixed as described above, followed by two washes in Perm-S buffer. Antibodies against intracellular targets were incubated with permeabilised cells in Perm-S buffer for 30 min at 4 °C. At the end of the staining, cells were washed twice in MaxPar staining buffer and stored in 1 ml of MaxPar Fix and Perm Buffer containing 125 nM MaxPar Intercalator-Ir (^191^Ir and ^193^Ir) at 4 °C. After 12 h, cells were washed twice in MaxPar staining buffer and stored as pellet in MaxPar staining buffer at 4 °C until analysis. To minimise the batch effect, samples were stained all in one batch then analysed by CyTOF in two sequential days (the day after cell staining). On the day of analysis, cells were washed twice in MaxPar water and re-suspended in MaxPar water containing 10% EQ™ four element calibration beads followed by acquisition on CyTOF.

### Data analysis

FCS files from CyTOF were normalised with calibration beads and concatenated by Helios Software v6.5.358, debarcoded by Fluidigm Debarcoder v1.04, and then submitted to Cytobank for gating and viSNE analysis. Manual gating was performed as shown in Additional file 1: Figure S2. CD45^+^ populations were used for viSNE analysis using default settings and with all markers except CD45 as annotation channels. T cell viSNE was conducted using default settings with total CD3 T cell populations and using CD4, CD8, CCR7, CD45RA, CD38, HLADR, CD69, and CD25 as the clustering channels. Pre-processed data files for our experiments can be downloaded from FlowRepository (FR-FCM-Z25H).

### Identification of cell subsets

FCS files of CD45^+^ cells were exported from Cytobank and submitted to automated phenotyping by X-shift algorithm using fast *k*-nearest-neighbour estimation from 150 (*k* = 150) to 5 (*k* = 5) neighbours for density estimate in 30 steps [31]. All markers except CD45 were used for clustering. Hierarchical clustering of these groups was performed using Euclidean distance and average linkage criterion. Spanning tree plots were generated with Euclidean distance in VorteX graphical environment with X-shift and associated visualisation tools incorporated in the software [31].

### Principal component analysis

Expression values of markers were *z*-score normalised and subjected to PCA analysis in R using prcomp() function. Scatter plots using the top two principal components are displayed.

### Statistics

Following cell subset identification, cell percentages were used for statistical analysis. Significance analyses were conducted using the Wilcoxon signed-rank test between PF and blood samples, Mann-Whitney *U* Test between control and endometriosis samples, and one-way ANOVA test among control, minimal/mild stage, and moderate/severe stage samples. Data were analysed using Prism 7 software (Graph Pad, Inc., San Diego, CA, USA).

## Results

### Phenotypic profiling of peritoneal fluid cells by mass cytometry

Major immune cell types including innate immune cells (such as cells of the mononuclear phagocytic system (MPS) including macrophages, monocytes, and dendritic cells (DCs), besides NK cells and neutrophils) and adaptive immune cells (T cells and B cells) have been associated with endometriosis; accordingly, we designed a panel of 33 antibodies that includes markers for the identification of these major cell types, in addition to markers that define their differentiation and plasticity states (Table 1 and Additional file 1: Table S1) [17, 32–34]. Peritoneal fluid cells (PFCs) and peripheral blood cells (PBCs) from endometriosis patients and controls free of endometriosis were collected during laparoscopic surgery, isotopically labelled, and processed for CyTOF acquisition and downstream data analysis (Additional file 1: Figure S1).

**Table 1:** Antibody panel list

Number	Marker	Protein	Typical target	Metal
1	CD117	Mast/stem cell growth factor receptor	Mast cells, ILCs, HSCs, CMPs	143Nd
2	CD38	Cyclic ADP-ribose hydrolase	Activated cells	144Nd
3	CD4	T cell surface glycoprotein CD4	T helper cells	145Nd
4	CD64	High affinity immunoglobulin gamma Fc receptor I	Monocytes/macrophage, M1 marker	146Nd
5	CD20	B lymphocyte antigen CD20	B cells	147Sm
6	CD16	Lymphocyte Fc gamma type III low-affinity receptor	Monocytes/macrophages, NK cells, neutrophils	148Nd
7	CD127	Interleukin-7 receptor-α	T cells, NK cells, ILCs	149Sm
8	CD40	Tumour necrosis factor receptor superfamily member 5	M1 macrophage marker, B cells, DCs	150Nd
9	CD123	Interleukin-3 receptor-α	Plasmacytoid DCs, basophils	151Eu
10	CD45RA	Isoform of CD45	Naïve/memory T cells	152Sm
11	FceRIα	High affinity IgE receptor subunit alpha	Mast cells, basophils, antigen presenting cells	153Eu
12	CD45	Protein tyrosine phosphatase, receptor type, C	All haematopoietic cells	154Sm
13	HLADR	HLA class II histocompatibility antigen DR	Monocytes/macrophages, DCs, B cells, NK cells	155Gd
14	CD69	Early activation antigen CD69	Early activation marker	156Gd
15	CD25	Interleukin-2 receptor-α	Treg cells, mature B cells	158Gd
16	CD11C	Integrin alpha-X	DCs, Monocytes/macrophages	159 Tb
17	CD14	Monocyte differentiation antigen CD14	Monocytes/macrophages, B cells	160Gd
18	Ki67	Proliferation marker protein Ki-67	Proliferating cells	161Dy
19	CD8	T cell surface glycoprotein CD8	Cytotoxic T cells	162Dy
20	CD27	Tumour necrosis factor receptor family member CD27	Activated T cells, naïve/memory B cells	163Dy
21	CCR7	C-C chemokine receptor type 7	Effector T cells	164Dy
22	CD163	Haemoglobin scavenger receptor	M2 macrophage marker	165Ho
23	CD24	Signal transducer CD24	B cells, granulocytes	166Er
24	GNLY	Granulysin	Cytolytic granules	167Er
25	CD206	Macrophage mannose receptor 1	M2 macrophage marker	168Er
26	NKG2A	Inhibitory NK cell receptor	NK cells, T cells	169Tm
27	CD3	T cell surface glycoprotein CD3	T cells	170Er
28	CD68	Macrosialin	Macrophages/monocytes	171Yb
29	CD9	Tetraspanin family member CD9	Haematopoietic cells	172Yb
30	KIR2DL2/3	Killer cell immunoglobulin-like receptor 2DL2/3	NK cells	173Yb
31	CD94	Killer cell lectin-like receptor subfamily D, member 1	NK cells, T cells	174Yb
32	CD11b	Integrin alpha-M	Monocytes/macrophages, neutrophils	175Lu
33	CD56	Neural cell adhesion molecule 1	NK cells	176Yb

*ILCs* innate lymphoid cells, *HSCs* haematopoietic stem cells, *CMPs* common myeloid progenitors

**Fig. 1:**
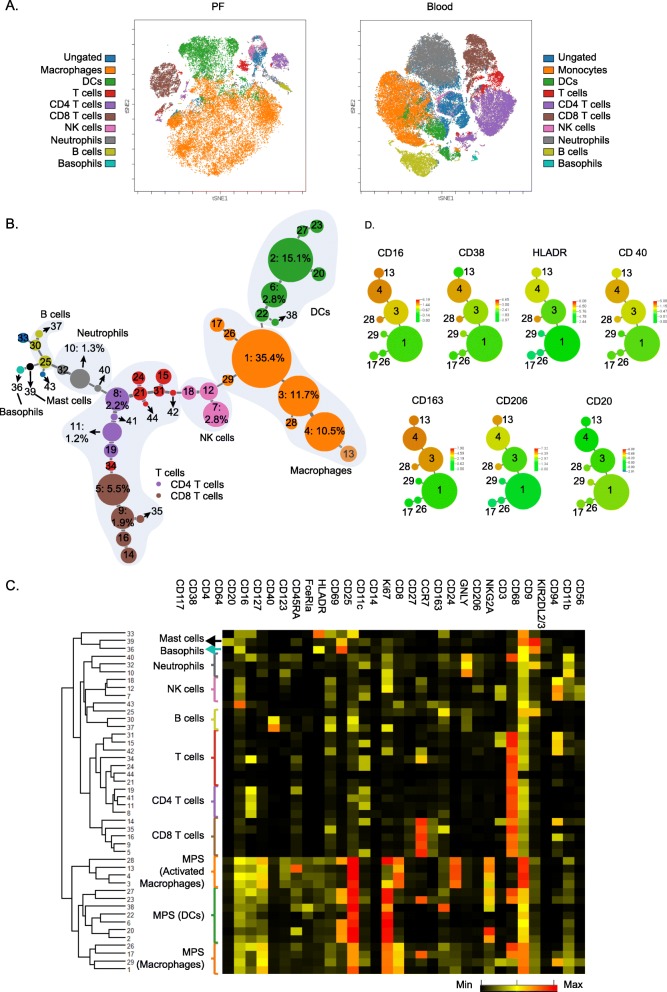
Peritoneal fluid cells show complex phenotypic heterogeneity. **a** viSNE plots showing the composite profiles of PFCs and PBCs. Haematopoietic cells from all PF (*n* = 20) and all blood (*n* = 20) samples were used for the analysis. Clouds of cells are generated by viSNE analysis. Each dot in the plots represents a single cell, and its colour suggests its immune cell type derived from manual gating (see Additional file 1: Table S3 and Additional file 1: Figure S2). **b** Phenotypic mapping of PFCs shown by minimum spanning tree plot. A composite plot of all PF samples is shown (plots from each sample are listed in Additional file 2). Each node represents a cell cluster, and node size indicates abundance of the cluster. X-shift algorithm identified 44 subpopulations (*k* = 40) that are named according to their ranking of proportions in all PFCs (from group 1 to group 44). Percentage in total cells of each group from group 1 to group 11 are labelled. Proportions of all other groups (group 12 to group 44) are below 1%. **c** Expression phenotypes of markers in these clusters are shown in the heat map (each row represents an individual cluster; numbers on the left indicate group names; black represents the minimum, yellow represents the median, and red represents the maximum expression value). These subpopulations were hierarchically clustered based on their marker expression patterns. **d** Spanning tree plots showing expression of activation markers on macrophage clusters. M1 and M2 activation markers are co-expressed on macrophages. A marker with negative expression (CD20) is also shown. Colour scales indicate intensities of markers. Group IDs are labelled in the plots

Our result shows that the majority of PFCs are immune cells (CD45^+^), in addition to a very small proportion of non-immune cells (CD45^−^) with an average of 1.85% in total cells (Additional file 1: Figure S2). To visualise the immune cell profiles of the peritoneal cavity, a viSNE analysis was first applied to CD45^+^ cells from PF and peripheral blood samples across both control and endometriosis donors. This neighbourhood embedding technique allows us to visualise groupings of cells based on the expression of all markers in both PFC and PBC samples [35]. Major cell subsets were also manually gated and annotated with different colours. We overlaid viSNE clouds with colours from manual gating (gating strategies are shown in Additional file 1: Table S3 and Additional file 1: Figure S2 and Figure S3). Correlations of viSNE clusters and colours derived from manual gating show profiles of cell major subsets from PF and blood (Fig. 1a). MPS members (macrophages and dendritic cells) constitute the largest proportion of cells in PFCs samples, when compared to PBCs. T cells, NK cells, B cells, and neutrophils were also detected, albeit to a much lower frequency. Taken together, we can clearly demonstrate that peritoneal immune profiles differ substantially from the circulating blood compartment. We also applied principal component analysis (PCA) on the expression values of all markers on CD45^+^ cells. We found a clear separation between PF and blood samples, with PBCs showing less variation when compared to PFCs (Additional file 1: Figure S4).

To further explore functional differences in immune subpopulations, data from CD45^+^ PFCs and PBCs were submitted to automated mapping of phenotypes using X-shift, an automated clustering algorithm that processes multidimensional single-cell data using fast *k*-nearest-neighbour estimation of cell event density [31]. This approach identified 44 clusters with distinct expression patterns of markers in PFCs that are shown in a minimum spanning tree (MST) plot (Fig. 1b; PBC result see Additional file 1: Figure S5; individual patient plots are listed in Additional file 2). These subpopulations were hierarchically clustered into major cell types in a heat map (Fig. 1c), demonstrating that these PFCs display a highly complex pattern, considerably expanding results from conventional flow cytometry studies where only a few markers could be investigated [36–38]. This approach easily distinguishes populations belonging to the MPS from lymphocyte systems (Fig. 1b). From these 44 cell groups, 11 distinct groups represent the largest fraction of PF haematopoietic cells (ranging from 1 to 24% of total cells). Within these major populations, cell groups 1–4 and 6 belong to the MPS system and make up the largest fraction (75%). Group 1 is a macrophage subpopulation [17]; groups 2 and 6 are DC-like cells expressing CD11c in addition to high FceRIa and CD206 expression [39], whereas groups 3 and 4 are activated macrophages (CD163^+^/CD206^+^) [17]. Among the remaining lymphocyte/leukocyte fractions of the major groups, 5 and 9 are activated CD8 T cells (CD69^+^/CD27^+^) [40, 41]; group 7 represents activated NK cells (CD69^+^) [42]; groups 8 and 11 are CD4 T cells, and group 10 is a neutrophil population [43]. Using this approach, rare populations were also identified such as mast cells (group 39) and basophils (group 33).

Interestingly, in addition to being alternatively activated (CD163^+^CD206^+^), macrophages of groups 3, 4, 13, and 28 also express CD16 and CD40 which are regarded as pro-inflammatory signatures (Fig. 1d) [44–46], indicating that they likely have undergone both M1 and M2 stimulus exposure during disease progression.

### Peritoneal immune cells are characterised by a distinctive marker profile compared to peripheral blood

**Fig. 2:**
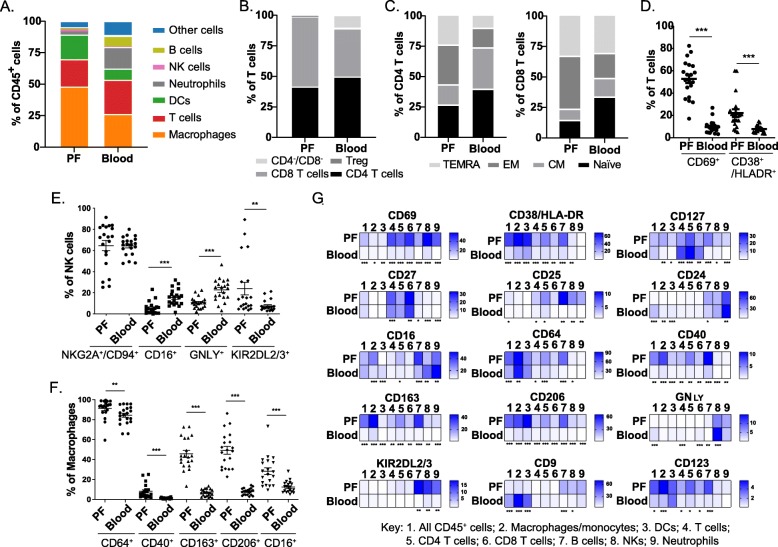
Immunological diversity and specialisation in PFCs. **a** Proportions of major cell populations in CD45^+^ cells show increased infiltration of macrophages, DCs, and NK cells in PF (*n* = 20) compared to blood (*n* = 20). Average proportions of cell subsets are shown (for patient-by-patient data, see Additional file 1: Figure S6A and S7). **b** Composition of T cell subsets from PF and blood shows increased CD8 T cells and decreased CD4 cells in PF (see Additional file 1: Figure S6B). **c** Percentages of naïve, CM, EM, and TEMRA as a proportion of total CD4 and CD8 T cells isolated form PF or blood suggest remarkably increased EM T cells in PF (see Additional file 1: Figure S6B). **d** Expression of CD69 and CD38/HLADR are increased on T cells from PF compared to blood. **e** NK cell cytotoxicity markers are reduced in PF. Frequencies of CD16^+^ and GNLY^+^ cells are higher in blood, while frequency of KIR2DL2/3^+^ cells is decreased in PF compared to blood NK cells. **f** CD64^+^, CD40^+^, CD163^+^, and CD206^+^ macrophages are significantly increased in PF. Means ± SEM are shown in scatter plots. **g** Heatmaps showing expression of activation markers in nine cell populations: 1, all CD45^+^ cells; 2, macrophages/monocytes; 3, DCs; 4, T cells; 5, CD4 T cells; 6, CD8 T cells; 7, B cells; 8, NKs; 9, Neutrophils. Scale bars indicate the mean percentages of marker expressing cells with respect to total cells in each population in PF (*n* = 20) compared to blood (*n* = 20) samples. Asterisks below each heatmap indicate the statistical significance. Wilcoxon’s signed-rank test was used in all statistics. *p < 0.05; **p < 0.01; ***p < 0.001

Immune phenotypes of PFCs and PBCs are distinct as shown by automated mapping (Fig. 1 and Additional file 1: Figure S5), making it difficult to compare phenotypic clusters between them. Therefore, we manually gated the subpopulations within CD45^+^ cells and compared their relative proportions. Abundances of macrophages, DCs, and NK cells were increased, whereas B cells and neutrophils were decreased in PF compared to peripheral blood (Fig. 2a, Additional file 1: Figure S6A and S7).

We looked at the major T cell subsets, CD4, CD8, and Treg cells (CD25^+^/CD127^−^; Fig. 2b, Additional file 1: Figure S6B), plus their differentiation states (naïve (CCR7^+^/CD45RA^+^), central memory (CM, CCR7^+^/CD45RA^−^), effector memory (EM, CCR7^−^/CD45RA^−^), and terminally differentiated effector memory (TEMRA, CCR7^−^/CD45RA^+^) T cells) (Fig. 2c, Additional file 1: Figure S6B) and activation (CD38^+^/HLADR^+^; Fig. 2d) [32, 47]. Compared to PBCs, relative frequencies of CD8 T cells, Treg cells, and effector (EM and TEMRA) T cells and a global activation of T cells in total were increased in PF, whereas reduced proportions of naïve and CM T cells were observed.

In PF, the cytotoxic NK cell subset (CD16^+^/CD56^dim^) and NK cells that express granulysin (GNLY^+^) were reduced (Fig. 2e), suggesting a reduced capacity for tissue clearance by NK cells. In addition, one major inhibitory killer immunoglobulin-like receptor (KIR2DL2/3^+^) was significantly (*p* ≤ 0.05) induced in NK PFCs, suggesting that NK cytotoxicity may be compromised in the peritoneal cavity (Fig. 2e).

As indicated above, we confirmed the previously noted alternative activation pattern of macrophages [13, 37], as shown by increased expression of CD163 and CD206 in PF (Fig. 2f). However, we also found pro-inflammatory signatures to be increased, including M1 markers CD64 and CD40 [17], as well as CD16, a marker for ‘non-classical’ macrophages, shown to display inflammatory features [45, 48].

Furthermore, we gated for expression of functional markers on subpopulations, showing global increases of inflammatory signatures such as CD69, CD38/HLADR, CD27, CD25, CD163, CD206, CD64, and CD40, and a decrease of cytotoxicity markers as shown by alterations of CD16, GNLY, KIR2DL2/3, and CD9 on NK cells, neutrophils, and T cell subsets (Fig. 2g).

### T cell expression of CD69 in PF is increased in endometriosis

**Fig. 3:**
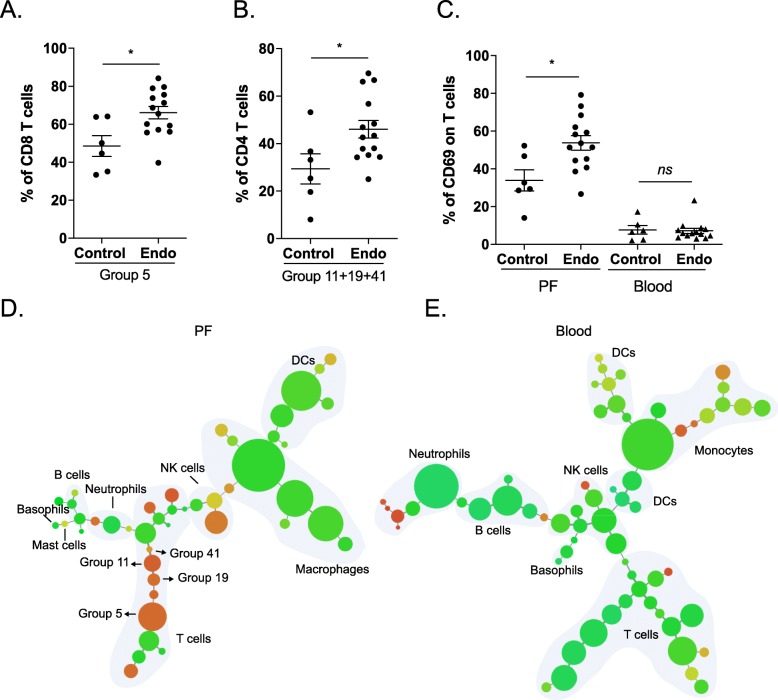
CD69 expression on T cells is increased in endometriosis PF. Abundances of group 5 in CD8 T cells (**a**) and combination of three groups, 11, 19, and 41, in CD4 T cells (**b**) are increased in endometriosis PF samples (*n* = 14) compared to controls (*n* = 6). Endo, endometriosis. **c** Stacked plots showing expression of T cell early activation markers, CD69, on T cells using manual gating suggest that CD69^+^ T cells are increased specifically in PF samples from endometriosis patients. **d**, **e** CD69 expression profiles in PF and blood samples. The spanning tree plots show hierarchies of all cell clusters in PF (**d**) and blood (**e**) samples generated from X-shift analysis. Each node represents a cell cluster, and node size indicates abundance of the cluster. Colour scales indicate intensities of CD69, suggesting that it is predominantly expressed on T cells in PFCs. Groups 5, 11, 19, and 41 from PFCs are labelled on tree plot. Means ± SEM are shown in scatter plots. The Mann-Whitney *U* test was used in all statistical calculations. *p < 0.05

In order to dissect the PF immune microenvironment further in endometriosis patients, we compared the percentiles of all 44 cell groups obtained from X-shift analysis in their corresponding immune subpopulations. From this analysis, two clusters were found to be significantly different between control and endometriosis PF samples (Fig. 3a, b). Group 5 and groups 11 + 19 + 41 constitute an enrichment of CD69^+^ CD4 and CD69^+^ CD8 T cells, respectively (Fig. 1b, c). We validated the expression of CD69 on T cells by manual gating, confirming a specific increase of CD69-expressing T cells in PF from endometriosis patients (Fig. 3c). In order to rule out that expression of CD69 is confounded by menstrual phase or hormone treatment, we investigated the effect of CD69 expression across each of the phases and treatments (two-way ANOVA). This revealed no significant effects of either hormone treatment or menstrual cycle phase on disease status (Additional file 1: Figure S8). To visualise the expression profile of CD69, we generated MST plots of clusters from X-shift analysis, which show that CD69 is indeed predominantly expressed on T cells in PFCs (Fig. 3d, e). Thus, our comparison of control and endometriosis samples reveals that the T cell activation marker CD69 is uniquely upregulated in PF samples. This suggests that CD69 is a novel and major signature correlated with endometriosis in the peritoneal environment.

### Other immune signatures associated with endometriosis and correlated with disease stages

**Fig. 4:**
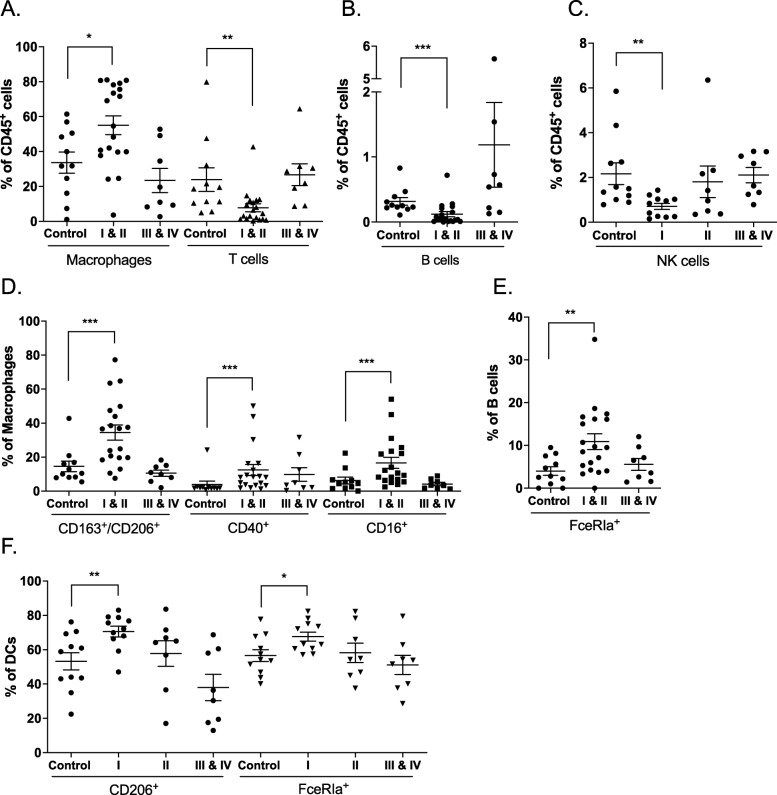
Immune signatures associated with endometriosis and correlated with disease stages. **a**–**c** Frequencies of macrophages and T cells (**a**), B cells (**b**), and NK cells (**c**) show significant differences at minimal/mild disease stages (see Additional file 1: Figure S9). **d** Alternative (CD163^+^/CD206^+^) and classical (CD40 and CD16) activation markers on macrophages are increased significantly at minimal/mild stages. **e** Abundances of FceRIa^+^ B cells are increased at minimal/mild disease stages. **f** Frequencies of CD206^+^ and FceRIa^+^ DCs at stage I are increased. Means ± SEM are shown. The Mann-Whitney *U* test was used in comparison between control and disease stage samples. *p < 0.05; **p < 0.01; ***p < 0.001. Control, *n* = 11; stage I, *n* = 11; stage II, *n* = 8; stages III and IV, *n* = 8

To identify additional differences between control and endometriosis samples, we measured a set of 38 PF samples with emphasis on monocyte/macrophage markers (Additional file 1: Table S4 and Table S5). After manual gating for major immune cell groups, we then analysed frequencies of functional markers across disease stages according to the widely used revised ASRM classification system [49]. Interestingly, immune alterations were found to be more dominant in minimal/mild disease stages (stage I and II) rather than in moderate/severe stages (stage III and IV). We detected increased macrophage infiltration and lower frequencies of T cells (Fig. 4a), B cells (Fig. 4b), and NK cells (Fig. 4c) in minimal/mild stages, which correlate with results derived from cell counts (Additional file 1: Figure S9A). Expanding the data described above, we found significantly increased frequencies of M2 (CD163/CD206) and M1 (CD40 and CD16) signatures in minimal/mild stages, which became reduced with more severe disease stages (Fig. 4d). The increased frequencies of FceRIa^+^ B cells (Fig. 4e) and FceRIa^+^ and CD206^+^ DCs (Fig. 4f) also appear to be restricted to minimal/mild disease stages (Fig. 4c). We examined the influence of menstrual phases and hormone treatment on the above results, showing that these factors do not confound the above results across disease stages (Additional file 1: Figure S9B and S9C). We also analysed data from PF and blood samples that were used in previous sections. After manual gating on major cell subsets, expression values of selected markers on these populations were extracted and PCA was performed. This revealed a separation on PC1 between endometriosis and control samples in PF, but not in blood (Additional file 1: Figure S10). This separation was driven in part by the expression of CD69, suggesting that CD69 may not be a suitable blood biomarker for endometriosis.

### CD69^+^ T cells in peritoneal fluid show decreased functional markers compared to peripheral blood cells

**Fig. 5:**
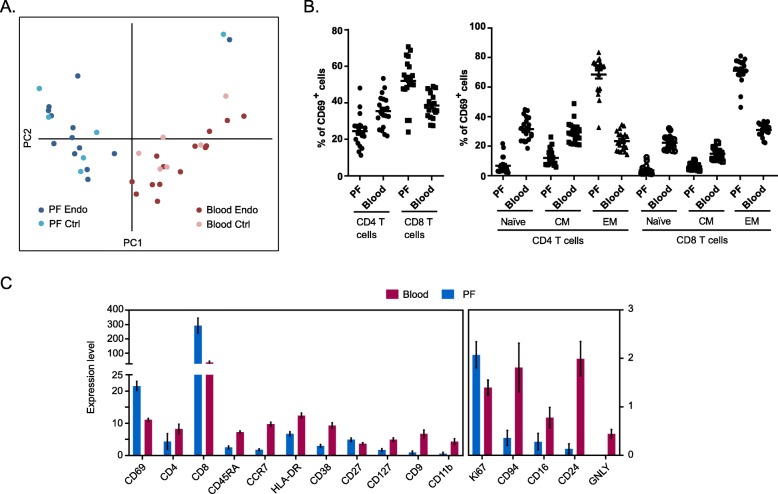
Comparison of CD69^+^ populations in PF and blood. **a** CD69^+^ T cells in PF and blood show distinct variation based on expression levels of all tested markers by PCA. **b** Composition of major subsets in CD69^+^ T cells. Compared to blood, CD69^+^ T cells in PF consist higher frequencies of CD8 and EM T cells. Means ± SEM are shown in plots. Statisitics were calculated by Wilcoxon’s signed-rank test and *p* < 0.001 for all comparisons. **c** Expression levels of markers that significantly differ in CD69^+^ T cells between PF and blood. Compared to blood counterpart, PF CD69^+^ T cells show reduced activation and functional activity. Means ± SEM are shown in plots. All comparisons between PF and blood showed significance (*p* < 0.05) by Wilcoxon’s signed-rank test

Given the specific increase of CD69 on PF T cells, we wanted to know if there is any difference on the CD69^+^ population in PF and blood from endometriosis and control samples. PCA of expression values of markers differentiates PF CD69^+^ cells from blood CD69^+^ cells, although control and endometriosis CD69^+^ cells are alike (Fig. 5a). An increase of CD8 and EM T cell frequencies and a decrease of CD4, naïve, and CM T cells were found in CD69^+^ PF T cells (Fig. 5b), whereas frequencies of Treg cells and TEMRA T cells do not change (data not shown). Moreover, comparison of marker expression levels in CD69^+^ cells from PFCs and PBCs showed that markers associated with cell activation (CD38 and HLADR) and cytotoxicity (GNLY and CD16) are reduced in CD69^+^ T cells from PFCs compared to PBCs (Fig. 5c). In addition, CD69^+^ PF T cells also failed to induce other functional markers, including CD9, CD11b, CD94, and CD24. Our findings suggest that although CD69^+^ cells in peritoneal fluid contain more effector memory CD8 cells, they may be less functionally active compared to blood counterparts.

### CD69 defines distinct phenotype and function in peritoneal fluid T cell lineages

**Fig. 6:**
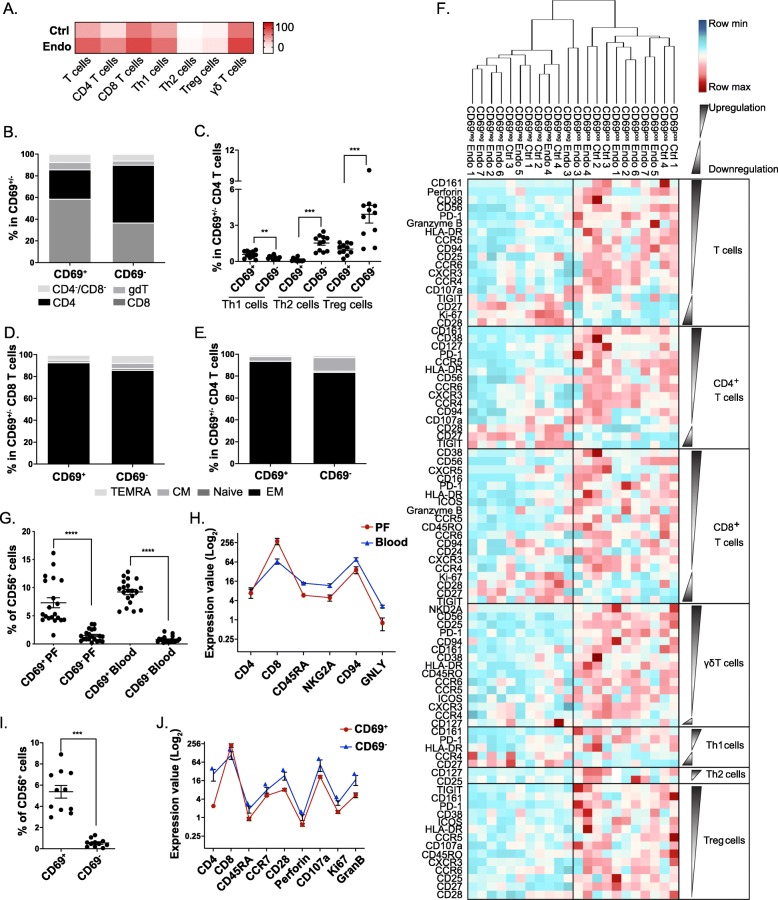
CD69 defines distinct phenotype across T cell lineages in PF. **a** Frequencies of CD69 across T cell lineages are generally increased on endometriosis (*n* = 7) PFCs compared to controls (*n* = 4) (see Additional file 1: Figure S11). **b**–**e** Comparison of subset composition between CD69^+^ and CD69^−^ PF T cells. **b** A stacked bar plot showing the frequency of PF CD4, CD8, and γδ T cells as a percentage of the total CD69^+^ or CD69^−^ T cells. **c** The frequency of Th1 cells, Th2 cells, and Treg cells as a percentage of total CD69^+/−^ cells. **d** A stacked bar plot showing the frequencies of CM, Naïve, EM, and TEMRA as a percentage of the total CD69^+^ or CD69^−^ CD8 T cells. **e** A stacked bar plot showing the frequencies of CM, Naïve, EM, and TEMRA as a percentage of the total CD69^+^ or CD69^−^ CD4 T cells. **f** Differentially expressed markers (*p* < 0.05) in subsets between CD69^+^ and CD69^−^ PF T cells shown by heat map for each sample. Samples were hierarchically clustered based on their marker expression patterns. **g**–**j** Comparison of CD56^+^ cells between CD69^+^ and CD69^−^ T cells from PF and blood. **g** Frequencies of the expression of CD56 on CD69^+^ and CD69^−^ T cells from PF (*n* = 20) and blood (*n* = 20) paired samples. **h** The expression of CD4, CD8, CD45RA, NKG2A, CD94, and GNLY in CD56^+^CD69^+^ T cells from PF and blood. **i** Comparison of CD56 expression on CD69^+^ and CD69^−^ cells from T cells sorted from PF samples (*n* = 11). **j** The expression of CD4, CD8, CD45RA, CCR7, CD28, Perforin, CD107a, KI67, and Granzyme B in CD56^+^CD69^+^ and CD56^+^CD69^−^ T cells. Means ± SEM are shown in plots

We next applied a T cell antibody panel (Additional file 1: Table S6) designated to investigate T cell subtypes and functions, and their association with CD69 on samples from additional patients of the same menstrual phase and without hormone treatment. T cells were sorted from PFCs from four control and seven stage I endometriosis samples (Additional file 1: Table S7) and analysed by CyTOF. Results show that the constitution of PF T cells includes naïve/memory CD4 and CD8 T cells, type 1 and type 2 T helper (Th1 and Th2) cells, Treg cells, and γδ T cells (TCRγδ^+^) (Additional file 1: Figure S11A). In line with the above findings, upregulation of CD69 on T cells was identified as a signature associated with endometriosis. Moreover, this increase occurs broadly across distinct T cell lineages (Fig. 6a and Additional file 1: Figure S11B). By comparing CD69^+^ population with CD69^−^ cells, we found increased frequencies of CD8 cells, γδ T cells, and Th1 cells (Fig. 6b, c), and increased EM T cells as a dominant population in CD4 and CD8 cells (Fig. 6d, e). We next compared the expression levels of functional markers on T cell subsets and found that whereas phenotype of CD69 populations in control and endometriosis samples was not differentiable, CD69^+^ cells show significantly distinct expression levels of these markers from CD69^−^ populations (Fig. 6f). The majority of markers tested were upregulated in CD69^+^ T cell subsets, including cell activation (CD38 and HLADR), cytotoxicity (Gran B, perforin, and CD107a), and chemotaxis (CCR5, CCR6, and CXCR3) markers. Of note, CD56 abundance was found to be increased on CD69^+^ populations (Fig. 6g, i). CD56^+^ T cells are considered to be NK-like T cells that possess high proliferative and cytolytic activities. We found that there was lower expression of activity markers in CD56^+^CD69^+^ T cells from PF than in their blood counterpart (Fig. 6h) and CD56^+^CD69^−^ PF T cells (Fig. 6j), suggesting an increased frequency but reduced cytolytic activity of NK-like T cells associated with CD69.

## Discussion

Our study advances the understanding of the immune microenvironment present within the peritoneal cavity in patients suffering endometriosis and highlights several points of importance. Firstly, the work identifies a significant pro-inflammatory microenvironment in the peritoneal innate cell compartment, including increased presence and activation of macrophages, dendritic cells, and NK cells. Furthermore, our results confirm that macrophages are quantitatively the major cell population found in the PF of endometriosis patients, with significantly increased M2 activation (CD163^+^/CD206^+^) [10, 13], especially in patients with mild to moderate disease. Our work builds upon these observations, highlighting that these macrophages also display pro-inflammatory signatures, such as increased CD16 and CD40 levels, pointing towards enrichment of a hybrid M1/M2 profile cells within PF. Indeed, the previously postulated M1/M2 macrophage polarisation paradigm has now been significantly revised, suggesting that macrophages are capable of adopting a phenotypic ‘switch’ between M1 and M2 activation depending on the microenvironment [50]. The importance of these macrophage phenotypes is emphasised through observations in mouse models where an M2 profile facilitates the growth of endometriotic lesions, as opposed to a pro-inflammatory M1 phenotype which protects from the disease [13]. Accordingly, targeting the reprogramming of macrophages has been proposed as a novel way to treat immune diseases [50, 51], which might be applicable to endometriosis.

Furthermore, we detect significant frequencies of cells displaying a DC-like phenotype (CD14^−^/CD11C^+^/HLADR^+^) in PFC, indicating an important role for DCs or DC-like cells in the peritoneal microenvironment. However, it is difficult at this stage to unambiguously assign these definitively as ‘dendritic cells’ based solely on the expression of markers such as CD11c. Indeed, a growing body of evidence demonstrates cellular plasticity between cells of the MPS, and accordingly, it seems more appropriate to consider macrophages and dendritic cells as cells that exist on a continuous and overlapping spectrum [52]. Our results support this view that DC populations in PFCs exert phenotypic similarities but are distinguishable from ‘classical’ macrophages. For example, we find previously unrecognised increases of FceRIa^+^ and CD206^+^ DC populations in the PF of endometriosis patients compared to controls. This complexity suggests further roles for DCs in endometriosis, besides established functions such as regulation of angiogenesis and immune cell activation during lesion development in endometriosis [53]. Interestingly, CD206 has been regarded as a differentiation marker of immature DCs [54], found within endometriotic lesions and the surrounding peritoneal membrane of women with endometriosis [55]. Expression of the immunoglobulin receptor FceRIa on DCs in endometriosis has not been reported; however, it is evidenced that FceRI-mediated DC antigen presentation leads to the development and activation of Th2 cells and antigen-specific T cell tolerance [56–58]. However, despite the clear presence of DCs in PF, their role in endometriosis is less clear, since mouse studies have suggested that DC depletion can either promote or attenuate endometriosis development [59, 60].

Previous studies have identified a clear link between endometriosis and the innate immune system, a finding that we have emulated in this study [61, 62]. Nevertheless, in our study, we also detected significant changes in the adaptive immune compartment, highlighted by the increased T cell activation and effector activity in PF. Expression of the pleiotropic immune activation marker CD69, a member of the C-type lectin superfamily [42], is specifically increased on T cells in PF, which we identified as a major T cell signature in the peritoneal environment associated with endometriosis. Although CD69 has been regarded as one of the earliest activation cell surface markers on leukocytes, research in CD69-deficient mice reveals that it may also be a negative regulator of autoimmune reactivity and inflammation in collagen-induced arthritis [63]. Moreover, Yanmei and colleagues identified induced CD69^+^CD4^+^CD25^−^ T cell population along with tumour progression in an orthotopic hepatic tumour mouse model. This T cell population was found to exert a regulatory function by suppressing T cell proliferation [64]. We detected decreases in the activation and functional activity of CD69^+^ PF T cells, when compared to the blood counterpart. Given that others have suggested a suppressive role for CD69^+^ T cells, this may indicate that these cells play an immunosuppressive role in the PF. Analysis of immune signatures on CD69^+^ CD4, CD69^+^ CD8, CD69^+^ Treg, and CD69^+^ γδ T cells revealed a similar expression pattern for functional markers, when compared to their respective CD69^−^ T cell subsets. Interestingly, increases of NK-like T cells (CD56^+^) in the CD69^+^ population may suggest a cytolytic role for these cells. However, our analysis showed that PF CD69^+^ NK-like T cells possess lower expression of cytolytic markers than in both CD69^+^ blood NK-like T cells and CD69^−^ PF NK-like T cells, suggesting that these CD69^+^ NK-like T cells from PF might be less effective in clearing endometrial fragments.

The significance of CD69 expression by T cells in endometriosis has not yet been determined. However, increased CD69 expression on CD56^+^ cells in PF from endometriosis has been reported [65]. In addition, CD161 was a top-increased signature associated with CD69 in our study. While CD161 is typically used to identify Th17 cells [66], it has also been used to define a distinct innate-like functional phenotype across T cell lineages [67]. Moreover, CD161^high^CD8^+^ T cells are pathogenetic in multiple sclerosis mouse models [68], which is a disease that women with endometriosis have an increased likelihood of developing [69]. Therefore, CD69 identifies T cells with a distinct phenotype across lineages in the peritoneal cavity associated with endometriosis. Understanding their roles will contribute to elucidating their immunopathology and enable potential therapeutic strategies in the disease.

Current treatments for endometriosis mainly treat the symptoms of disease and not the underlying causes of inflammation or disease pathogenesis. Surgery has been shown to be effective; however, it can be associated with significant negative side effects in some women. Thus, if we are to develop new therapies towards endometriosis, new therapeutic targets are required. Our findings show that patients with endometriosis have a specific pro-inflammatory peritoneal immune microenvironment, including altered frequencies of both the innate and adaptive immune system. The application of mass cytometry to profile the inflammatory microenvironment has allowed us to better understand the immunopathogenesis of endometriosis and develop immunotherapy targets. However, it is not completely understood if the dysfunctional immune response is one of the triggers in endometriosis or a consequence that arises after the disease has developed. It has been observed that retrograde menstruation leads to innate immune activation, including increased numbers of macrophages, which is likely the first important step in the pathophysiology of endometriosis [68]. Our results demonstrate stage-dependent immune cell changes and adaptations in endometriosis and, therefore, offer a possible approach to improving the classification of this condition. Moreover, the use of mass cytometry and antibody panels designed in this study constitutes a potential improvement in the application as diagnostic tool in therapy development and precision medicine.

There are several limitations that should be noted for this study. Firstly, due to the sample size used in this study, we could not statistically analyse samples stratified by hormone treatments or menstruation phases. Although we identified a number of immune alterations on peritoneal cells without being confounded by menstruation phases or hormones, further larger studies should be undertaken to analyse changes stratified for these factors as well as disease stages. In larger cohorts, cycle phases and hormone treatment could be confounders and may need to be accounted for. Furthermore, due to the complexity of endometriosis, disease subtypes (including superficial peritoneal, cystic ovarian and deep endometriosis) in combination with the above conditions should be considered in future studies. Secondly, although we managed to capture a large number of immune cell subtypes, additional antibody panels could be applied for an even deeper immune characterisation, possibly complemented by single-cell transcriptomics. Moreover, intracellular phenotyping makers could be used, such as FOXP3 to define Treg cells and cytokines to define T helper subsets. Finally, with regard to the phenotypic changes found in this work, future functional validations are necessary, such as direct cytokine measurements or cytotoxicity assays. Undoubtedly, considering the immune complexity and dynamics of the disease, better understanding of the functional contribution to disease for each immune cell will help to explain the pathogenic mechanisms involved in endometriosis. Improvement of profiling tools and application of single-cell technologies as performed in this study are first steps towards this goal.

## Conclusions

In this study, mass cytometry was used to investigate immune cell compartments in endometriosis, revealing a heterogeneous and inflammatory microenvironment that is more complex than previously understood. We found PF-specific endometriosis-associated immune signatures from both innate and adaptive immune lineages, underlined by distinct phenotypes that were marked by CD69. In addition, our study also showed a dynamic spectrum of cell signatures across disease stages. Taken together, our findings provide a resource of peritoneal immune changes for future studies and suggest to employ CyTOF-based approaches to further the understanding of endometriosis pathogenesis and to potentially identify novel therapeutic strategies.

## Supplementary information

**Additional file 1: **Lists of antibody and patients information used in this study and additional figures in support of the mass cytometry analysis. **Table S1.** Related to Fig. 1 and Experimental Procedures. Details of antibodies used in this study. **Table S2.** Related to Fig. 1. Information of 20 patients from whom PF and blood are used in this study. **Table S3.** Related to Fig. 1, Fig. 2 and Fig. 6. Markers used to define immune cell types. **Table S4.** Related to Fig. 4 and Experimental Procedures. Antibodies used in the analysis of 38 PF samples. **Table S5.** Related to Fig. 4. Information of 38 patients in follow up study. **Table S6.** Related to Fig. 6 and Experimental Procedures. Antibodies used in the T cell panel. **Table S7.** Related to Fig. 6. Information of 11 patient samples used in T cell panel study. **Figure S1.** Graphic workflow of CyTOF study comparing PFCs and PBCs. **Figure S2 and Figure S3.** Related to Fig. 1, Fig. 2 and Fig. 6. Manual gating of cells subsets and functional markers. **Figure S4.** Related to Fig. 1. Clustering of PF and blood samples by PCA. **Figure S5.** Related to Fig. 1. Phenotypic mapping of PBCs. **Figure S6.** Related to Fig. 2. Percentage of major immune cells types in blood and PF samples and expression of functional markers. **Figure S7.** Related to Fig. 2. Cell counts show changes of major cell populations in PF compared to peripheral blood. **Figure S8.** Related to Fig. 3. Differential expression of CD69 in endometriosis was not affected by menstruation or hormone. **Figure S9.** Related to Fig. 4. Cell counts of major cell subtypes in PFCs at disease stages and evaluation of confounding effects from menstrual cycle and hormones. **Figure S10.** Related to Fig. 4. A. PCA separates endometriosis (Endo) and control in PF but not blood samples. **Figure S11.** Related to Fig. 6. ViSNE plot showing composition of T cells and comparison of CD69 abundance on T cell lineages between control and endometriosis samples from PF.

**Additional file 2.** Related to Fig. 1. Patient-by-patient minimum spanning tree plots showing cell clustering of PF and blood samples.

## Data Availability

Data supporting the findings of this study are available in supplementary information. Original mass cytometry data are available from the corresponding author upon reasonable request.

## Abbreviations

CM: Central memory
CyTOF: Mass cytometry
DCs: Dendritic cells
EM: Effector memory
M1 macrophages: Classically activated macrophages
M2 macrophages: Alternatively activated macrophages
MPS: Mononuclear phagocytic system
NK cells: Natural killer cells
PBCs: Peripheral blood cells
PCA: Principal component analysis
PFCs: Peritoneal fluid cells
TEMRA: Terminally differentiated effector memory
Th1 cells: Type 1 T helper cells
Th2 cells: Type 2 T helper cells
Treg cells: Regulatory T cells
