# Supplementary material for: Mass cytometry analysis reveals a distinct immune environment in peritoneal fluid in endometriosis: a characterisation study
Source: BMC Med. 2020 Jan 7;18:3. doi: 10.1186/s12916-019-1470-y (PMC6945609; doi:10.1186/s12916-019-1470-y)
Supplement: Supplementary file 1 — Additional file 1: Lists of antibody and patients information used in this study and additional figures in support of the mass cytometry analysis. Table S1. Related to Fig. 1 and Experimental Procedures. Details of antibodies used in this study. Table S2. Related to Fig. 1. Information of 20 patients from whom PF and blood are used in this study. Table S3. Related to Fig. 1, Fig. 2 and Fig. 6. Markers used to define immune cell types. Table S4. Related to Fig. 4 and Experimental Procedures. Antibodies used in the analysis of 38 PF samples. Table S5. Related to Fig. 4. Information of 38 patients in follow up study. Table S6. Related to Fig. 6 and Experimental Procedures. Antibodies used in the T cell panel. Table S7. Related to Fig. 6. Information of 11 patient samples used in T cell panel study. Figure S1. Graphic workflow of CyTOF study comparing PFCs and PBCs. Figure S2 and Figure S3. Related to Fig. 1, Fig. 2 and Fig. 6. Manual gating of cells subsets and functional markers. Figure S4. Related to Fig. 1. Clustering of PF and blood samples by PCA. Figure S5. Related to Fig. 1. Phenotypic mapping of PBCs. Figure S6. Related to Fig. 2. Percentage of major immune cells types in blood and PF samples and expression of functional markers. Figure S7. Related to Fig. 2. Cell counts show changes of major cell populations in PF compared to peripheral blood. Figure S8. Related to Fig. 3. Differential expression of CD69 in endometriosis was not affected by menstruation or hormone. Figure S9. Related to Fig. 4. Cell counts of major cell subtypes in PFCs at disease stages and evaluation of confounding effects from menstrual cycle and hormones. Figure S10. Related to Fig. 4. A. PCA separates endometriosis (Endo) and control in PF but not blood samples. Figure S11. Related to Fig. 6. ViSNE plot showing composition of T cells and comparison of CD69 abundance on T cell lineages between control and endometriosis samples from PF. [file 12916_2019_1470_MOESM1_ESM.pdf]

Supplemental Tables and Figures

Table S1. Related to Figure 1 and Experimental Procedures. Details of antibodies used in this study. Working volume and concentration represent the volume of the antibody added per 100 µl of staining buffer and the final concentration of antibody used for staining, respectively.

No	MARKER	Metal	Working vol. or conc.	Clone	Cat #	Company	Location
1	CD117	143Nd	1.5 µl	104D2	3143001B	Fluidigm	Cell surface
2	CD38	144Nd	1 µl	HIT2	3144014B	Fluidigm	Cell surface
3	CD4	145Nd	1 µl	RPA-T4	3145001B	Fluidigm	Cell surface
4	CD64	146Nd	1 µl	10.1	3146006B	Fluidigm	Cell surface
5	CD20	147Sm	1.2 µl	2H7	3147001B	Fluidigm	Cell surface
6	CD16	148Nd	1.5 µl	3G8	3148004B	Fluidigm	Cell surface
7	CD127	149Sm	1.5 µl	A019D5	3149011B	Fluidigm	Cell surface
8	CD40	150Nd	1 µg/ml	5C3	334325	BioLegend	Cell surface
9	CD123	151Eu	1.5 µl	6H6	151001B	Fluidigm	Cell surface
10	CD45RA	152Sm	1 µg/ml	HI100	304143	BioLegend	Cell surface
11	FcεRIα	153Eu	1 µg/ml	AERU37	334602	BioLegend	Cell surface
12	CD45	154Sm	0.5 µl	HI30	3154001B	Fluidigm	Cell surface
13	HLADR	155Gd	1 µg/ml	L243	307651	BioLegend	Cell surface
14	CD69	156Gd	2 µg/ml	FN50	310939	BioLegend	Cell surface
15	CD25	158Gd	2 µg/ml	M-A251	356102	BioLegend	Cell surface
16	CD11C	159Tb	1 µl	Bu15	3159001B	Fluidigm	Cell surface
17	CD14	160Gd	1 µl	M5E2	3160001B	Fluidigm	Cell surface
18	Ki67	161Dy	1.5 µl	B56	3161007B	Fluidigm	Intracellular
19	CD8	162Dy	1 µl	RPAUT8	3162015B	Fluidigm	Cell surface
20	CD27	163Dy	1 µg/ml	O323	302839	BioLegend	Cell surface
21	CCR7	164Dy	2 µg/ml	G043H7	353237	BioLegend	Cell surface
22	CD163	165Ho	1.5 µl	GHI/61	3165017B	Fluidigm	Cell surface
23	CD24	166Er	1 µl	ML5	3166007B	Fluidigm	Cell surface
24	Granulysin	167Er	2 µg/ml	DH2	348008	BioLegend	Intracellular
25	CD206	168Er	1 µl	15.2	3168008B	Fluidigm	Cell surface
26	NKG2A	169Tm	1.5 µl	Z199	3169013B	Fluidigm	Cell surface
27	CD3	170Er	1 µl	UCHT1	3170001B	Fluidigm	Cell surface
28	CD68	171Yb	1 µl	Y1/82A	3171011B	Fluidigm	Intracellular
29	CD9	172Yb	1.2 µl	SN4 C3U3A2	3172010B	Fluidigm	Cell surface
30	KIR2DL2/L3	173Yb	1.5 µl	DX27	3173010B	Fluidigm	Cell surface
31	CD94	174Yb	1.2 µl	HPU3D9	3174015B	Fluidigm	Cell surface
32	CD11b	175Lu	0.5 µg/ml	ICRF44	101249	BioLegend	Cell surface
33	CD56	176Yb	1.5 µl	N901	3176009B	Fluidigm	Cell surface

Table S2. Related to Figure 1. Information of 20 patients from whom PF and blood are used in this study.

ID	Disease Stage	Hormone treatment	Menstrual Cycle	Age
1	Control	No	Follicular Phase	23
2	Control	No	Follicular Phase	34
3	Control	No	Luteal Phase	45
4	Control	No	Luteal phase	22
5	Control	Yes	Irregular	22
6	Control	Yes	Unknown	25
7	Stage I	No	Irregular	28
8	Stage I	No	Irregular	29
9	Stage I	Yes	Menstruation	20
10	Stage I	Yes	No Periods	44
11	Stage I	Yes	Unknown	23
12	Stage II	No	Follicular phase	31
13	Stage II	Yes	Unknown	28
14	Stage III	No	Luteal Phase	33
15	Stage III	Yes	Irregular	24
16	Stage III	Yes	No Periods	34
17	Stage IV	No	Follicular Phase	45
18	Stage IV	No	Follicular phase	35
19	Stage IV	No	Luteal Phase	40
20	Stage IV	Yes	Irregular	30

Table S3. Related to Figure 1, Figure 2 and Figure 6. Markers used to define immune cell types.

**Cell types**	**Subtypes**	**Phenotypic markers**
Hematopoietic cells		$CD45^{+}$
Macrophages		$CD45^{+}$ $CD14^{+}$ $CD68^{high}$ $CD3^{-}$ $CD20^{-}$
	M1 macrophages	$CD40^{+}$ $CD64^{+}$
	M2 macrophages	$CD163^{+}$ $CD206^{+}$
DCs		$CD45^{+}$ $HLADR^{+}$ $CD11c^{+}$ $CD14^{-}$ $CD3^{-}$ $CD20^{-}$
T cells		$CD45^{+}$ $CD3^{+}$ $CD14^{-}$
	CD4 T cells	$CD4^{+}CD8^{-}$
	CD8 T cells	$CD8^{+}$ $CD4^{-}$
	Naïve T cells	$CCR7^{+}$ $CD45RA^{+}$
	CM T cells	$CCR7^{+}$ $CD45RA^{-}$
	EM T cells	$CCR7^{-}$ $CD45RA^{-}$
	TEMRA T cells	$CCR7^{-}$ $CD45RA^{+}$
	Treg	$CD4^{+}$ $CD25^{+}$ $CD127^{-}$
	Th1 cells	$CD4^{+}$ $CCR5^{+}$ $CXCR3^{+}$ $CCR4^{-}$ $CD25^{-}$
	Th2 cells	$CD4^{+}$ $CCR4^{+}$ $CCR5^{-}$ $CCR6^{-}$ $CXCR3^{-}$ $CD25^{-}$
B cells		$CD45^{+}$ $CD20^{+}$ $CD3^{-}$ $CD14^{-}$
NK cells		$CD45^{+}$ $CD56^{+}$ $CD14^{-}$ $CD3^{-}$ $CD20^{-}$
	Cytotoxic NK cells	$CD16^{+}$ $CD56^{dim}$
Neutrophils		$CD45^{dim}$ $CD11b^{+}$ $CD24^{+}$
Basophils		$CD45^{dim}$ $CD123^{high}$ $CD38^{high}$ $CD9^{+}$ $CD25^{dim}$ $HLADR^{-}$
Mast cells		$CD45^{dim}$ $CD117^{+}$ $FceRIa^{+}$

Table S4. Related to Figure 4 and Experimental Procedures. Antibodies used in the analysis of 38 PF samples. Working volume and concentration represent the volume of the antibody added per 100 µl of staining buffer and the final concentration of antibody used for staining, respectively.

No	MARKER	Metal	Working vol. or conc.	Clone	Cat #	Company	Location
1	CD117	143Nd	1.5 µl	104D2	3143001B	Fluidigm	Cell surface
2	CD4	145Nd	1 µl	RPA-T4	3145001B	Fluidigm	Cell surface
3	CD64	146Nd	1 µl	10.1	3146006B	Fluidigm	Cell surface
4	CD20	147Sm	1.2 µl	2H7	3147001B	Fluidigm	Cell surface
5	CD16	148Nd	1.5 µl	3G8	3148004B	Fluidigm	Cell surface
6	CD40	150Nd	1 µg/ml	5C3	334325	BioLegend	Cell surface
7	Fce RIα	153Eu	1 µg/ml	AERU37 (CRAU1)	334602	BioLegend	Cell surface
8	CD45	154Sm	0.5 µl	HI30	3154001B	Fluidigm	Cell surface
9	HLADR	155Gd	1 µg/ml	L243	307651	BioLegend	Cell surface
10	CD11C	159Tb	1 µl	Bu15	3159001B	Fluidigm	Cell surface
11	CD14	160Gd	1 µl	M5E2	3160001B	Fluidigm	Cell surface
12	CD8	162Dy	1 µl	RPAUT8	3162015B	Fluidigm	Cell surface
13	CD163	165Ho	1.5 µl	GHI/61	3165017B	Fluidigm	Cell surface
14	CD206	168Er	1 µl	15.2	3168008B	Fluidigm	Cell surface
15	NKG2A	169Tm	1.5 µl	Z199	3169013B	Fluidigm	Cell surface
16	CD3	170Er	1 µl	UCHT1	3170001B	Fluidigm	Cell surface
17	CD68	171Yb	1 µl	Y1/82A	3171011B	Fluidigm	Intracellular
18	CD9	172Yb	1.2 µl	SN4 C3U3A2	3172010B	Fluidigm	Cell surface
19	KIR2DL2/L3	173Yb	1.5 µl	DX27	3173010B	Fluidigm	Cell surface
20	CD94	174Yb	1.2 µl	HPU3D9	3174015B	Fluidigm	Cell surface
21	CD11b	175Lu	0.5 µg/ml	ICRF44	101249	BioLegend	Cell surface
22	CD56	176Yb	1.5 µl	N901	3176009B	Fluidigm	Cell surface

Table S5. Related to Figure 4. Information of 38 patients in follow up study.

ID	Disease Stage	Hormone treatment	Menstrual Cycle	Age
1	Control	No	Follicular	29
2	Control	No	Follicular	33
3	Control	No	Luteal	32
4	Control	No	Luteal	18
5	Control	Yes	Menstruation	41
6	Control	Yes	Irregular/no cycle	29
7	Control	Yes	Amenorrhea/unknown	20
8	Control	Yes	No periods	28
9	Control	Yes	Pill	24
10	Control	Yes	Pill/no cycle	25
11	Control	Yes	Unknown	36
12	Stage I	No	Follicular	22
13	Stage I	No	Follicular	34
14	Stage I	No	Follicular	28
15	Stage I	No	Follicular	36
16	Stage I	No	Luteal/ovulation	26
17	Stage I	No	Menstruation	41
18	Stage I	No	Menstruation	31
19	Stage I	No	Menstruation/Luteal	40
20	Stage I	No	Unknown	25
21	Stage I	Yes	Unknown	26
22	Stage I	Yes	Luteal/pill effect	30
23	Stage II	No	Follicular	32
24	Stage II	No	Follicular	36
25	Stage II	No	Irregular	34
26	Stage II	No	Luteal	44
27	Stage II	No	Menstruation	27
28	Stage II	No	Menstruation	32
29	Stage II	Yes	No periods	33
30	Stage II	Yes	Pill	24
31	Stage III	No	Follicular/Luteal	38
32	Stage III	No	Menstruation	21
33	Stage IV	No	Luteal	42
34	Stage IV	No	Luteal	45
35	Stage IV	No	Luteal/Follicular	43
36	Stage IV	Yes	Pill/Luteal	24
37	Stage IV	Yes	Tissue unsuitable	34
38	Stage IV	Unknown	Luteal	37

Table S6. Related to Figure 6 and Experimental Procedures. Antibodies used in the T cell panel.

No	MARKER	Metal	Working vol. or conc.	Clone	Cat #	Company	Location
1	CD45	Y89	0.5 µl	HI30	3089003B	Fluidigm	Cell surface
2	CCR6	141Pr	0.7 µl	G034E3	3141003A	Fluidigm	Cell surface
3	CD94	142Nd	1 µg/ml	DX22	305502	BioLegend	Cell surface
4	CD45RA	143Nd	0.8 µl	HI100	3143006B	Fluidigm	Cell surface
5	CCR5	144Nd	1 µl	NP-6G4	3144007A	Fluidigm	Cell surface
6	CD4	145Nd	0.5 µl	RPA-T4	3145001B	Fluidigm	Cell surface
7	CD8	146Nd	0.5 µl	RPA-T8	3146001B	Fluidigm	Cell surface
8	perforin	147Sm	2 µg/ml	dG9	308102	BioLegend	Intracellular
9	CD16	148Nd	0.8 µl	3G8	3148004B	Fluidigm	Cell surface
10	CD127	149Sm	0.8 µl	A019D5	3149011B	Fluidigm	Cell surface
11	LAG3	150Nd	0.6 µl	11C3C65	3150030B	Fluidigm	Cell surface
12	CD107a	151Eu	0.5 µl	H4A3	3151002B	Fluidigm	Cell surface
13	TCRγδ	152Sm	0.5 µl	11F2	3152008B	Fluidigm	Cell surface
14	TIGIT	153Eu	0.5 µl	MBSA43	3153019B	Fluidigm	Cell surface
15	HLADR	155Gd	0.5 µg/ml	L243	307651	BioLegend	Cell surface
16	CD69	156Gd	1 µg/ml	FN50	310939	BioLegend	Cell surface
17	CD25	158Gd	1 µg/ml	M-A251	356102	BioLegend	Cell surface
18	CD161	159Tb	0.8 µl	HP-3G10	3159004B	Fluidigm	Cell surface
19	CD28	160Gd	0.7 µl	CD28.2	3160003B	Fluidigm	Cell surface
20	Ki67	161Dy	1 µl	B56	3161007B	Fluidigm	Intracellular
21	CD27	162Dy	0.5 µl	L128	3162009B	Fluidigm	Cell surface
22	CXCR3	163Dy	0.5 µl	G025H7	3163004B	Fluidigm	Cell surface
23	CCR7	164Dy	1 µg/ml	G043H7	353237	BioLegend	Cell surface
24	CD45RO	165Ho	0.7 µl	UCHL1	3165011B	Fluidigm	Cell surface
25	CD24	166Er	1 µl	ML5	3166007B	Fluidigm	Cell surface
26	CD38	167Er	0.7 µl	HIT2	3167001B	Fluidigm	Cell surface
27	ICOS	168Er	1 µl	C398.4A	3168024B	Fluidigm	Cell surface
28	NKG2A	169Tm	1 µl	Z199	3169013B	Fluidigm	Cell surface
29	CD3	170Er	0.5 µl	UCHT1	3170001B	Fluidigm	Cell surface
30	CXCR5	171Yb	1 µl	RF8B2	3171014B	Fluidigm	Cell surface
31	Granzyme B	173Yb	0.5 µl	GB11	3173006B	Fluidigm	Intracellular
32	PD1	174Yb	1 µl	EH12.2H7	3174020B	Fluidigm	Cell surface
33	CCR4	175Lu	0.5 µl	L291H4	3175035A	Fluidigm	Cell surface
34	CD56	176Yb	0.8 µl	N901	3176009B	Fluidigm	Cell surface
35	CD11b	209Bi	0.5 µl	ICRF44	3209003B	Fluidigm	Cell surface

Table S7. Related to Figure 6. Information of 11 patient samples used in T cell panel study.

ID	Disease stage	Hormone treatment	Menstrual Cycle	Age
**1**	Control	No	Luteal	35
**2**	Control	No	Luteal	39
**3**	Control	No	Luteal	32
**4**	Control	No	Luteal	23
**5**	Stage I	No	Luteal	24
**6**	Stage I	No	Luteal	25
**7**	Stage I	No	Luteal	45
**8**	Stage I	No	Luteal	34
**9**	Stage I	No	Luteal	32
**10**	Stage I	No	Luteal	33
**11**	Stage I	No	Luteal	36

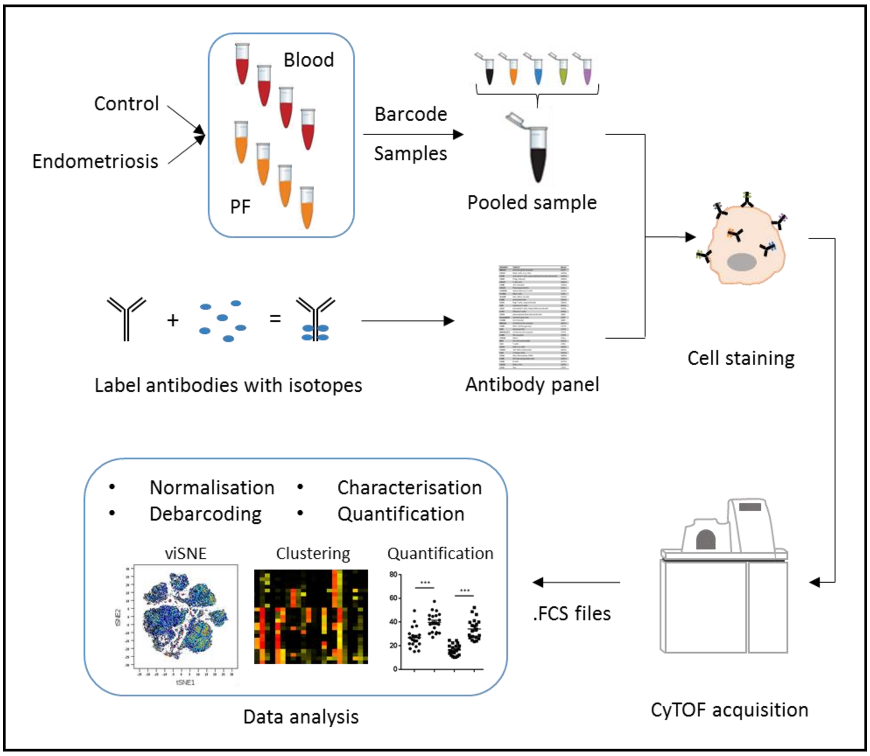

Figure S1. Graphic workflow of CyTOF study comparing PFCs and PBCs. Cells from matched peritoneal fluid and peripheral blood samples from endometriosis patients and non-endometriosis controls were collected and barcoded. Barcoded samples were pooled and stained using validated antibody panel followed by data acquisition on a CyTOF analyser. Results were analysed by a series of data analysis tools.

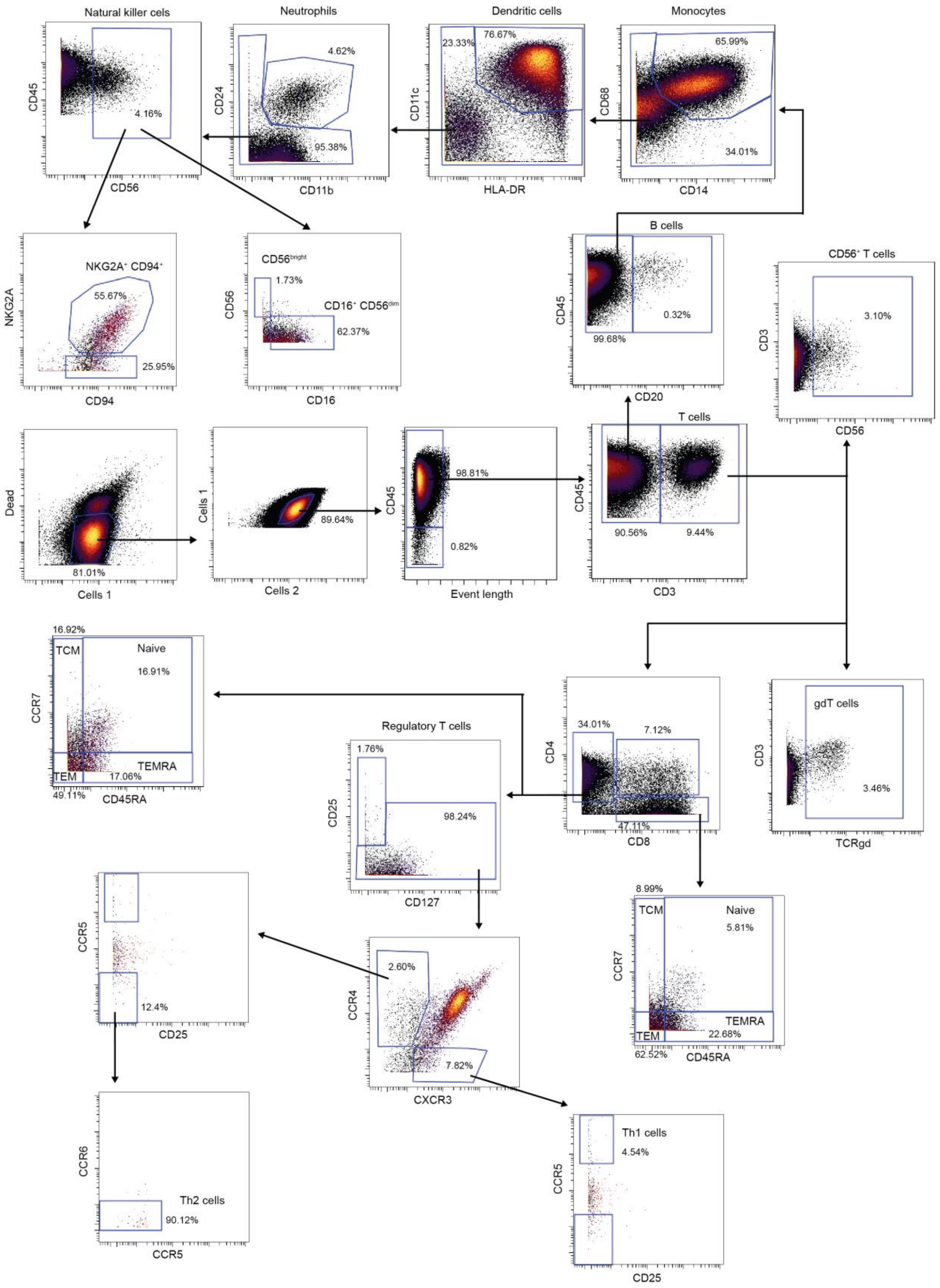

Figure S2. Related to Figure 1, Figure 2 and Figure 6. Manual gating of cells subsets from PFCs. Cells were gated against dead cell marker (intercalator-103Rh) and cell-ID intercalator 1 (^{191}Ir) for live cells, from which singlets were gated using cell-ID intercalator 1 (^{191}Ir) and intercalator 2 (^{193}Ir). Then hematopoietic cells and immune subtypes were gated using corresponding markers.

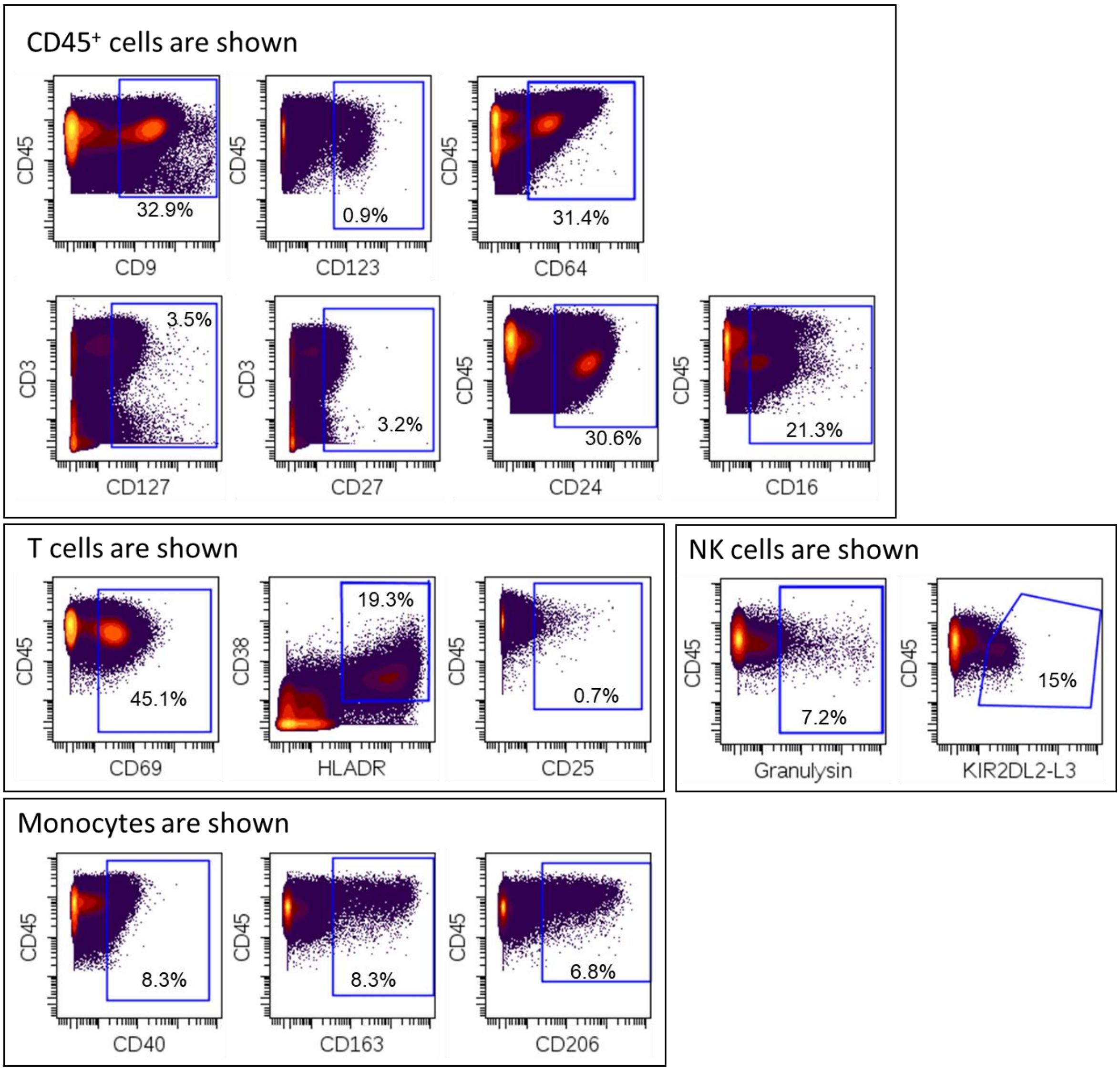

Figure S3: Dot plots showing the expression of several functional markers on CD45⁺ cells, T cells and NK and monocytes. Cell populations are determined using the method shown in Figure S2. Gates were determined either on all CD45⁺ cells or on subsets where they are preferably expressed (T cells, monocytes and NK cells).

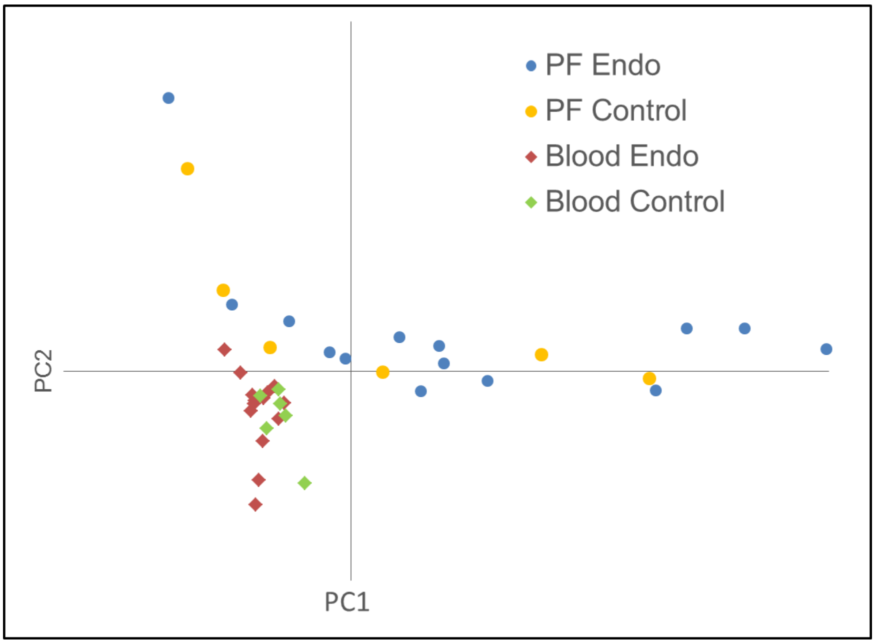

Figure S4. Related to Figure 1. Clustering of PF and blood samples by PCA. PCA was performed using expression values of all markers on $CD45^{+}$ cells, showing distinct distribution of PF and blood samples. Samples were plotted against the first two principal components (PC1 and PC2). Endo, endometriosis.

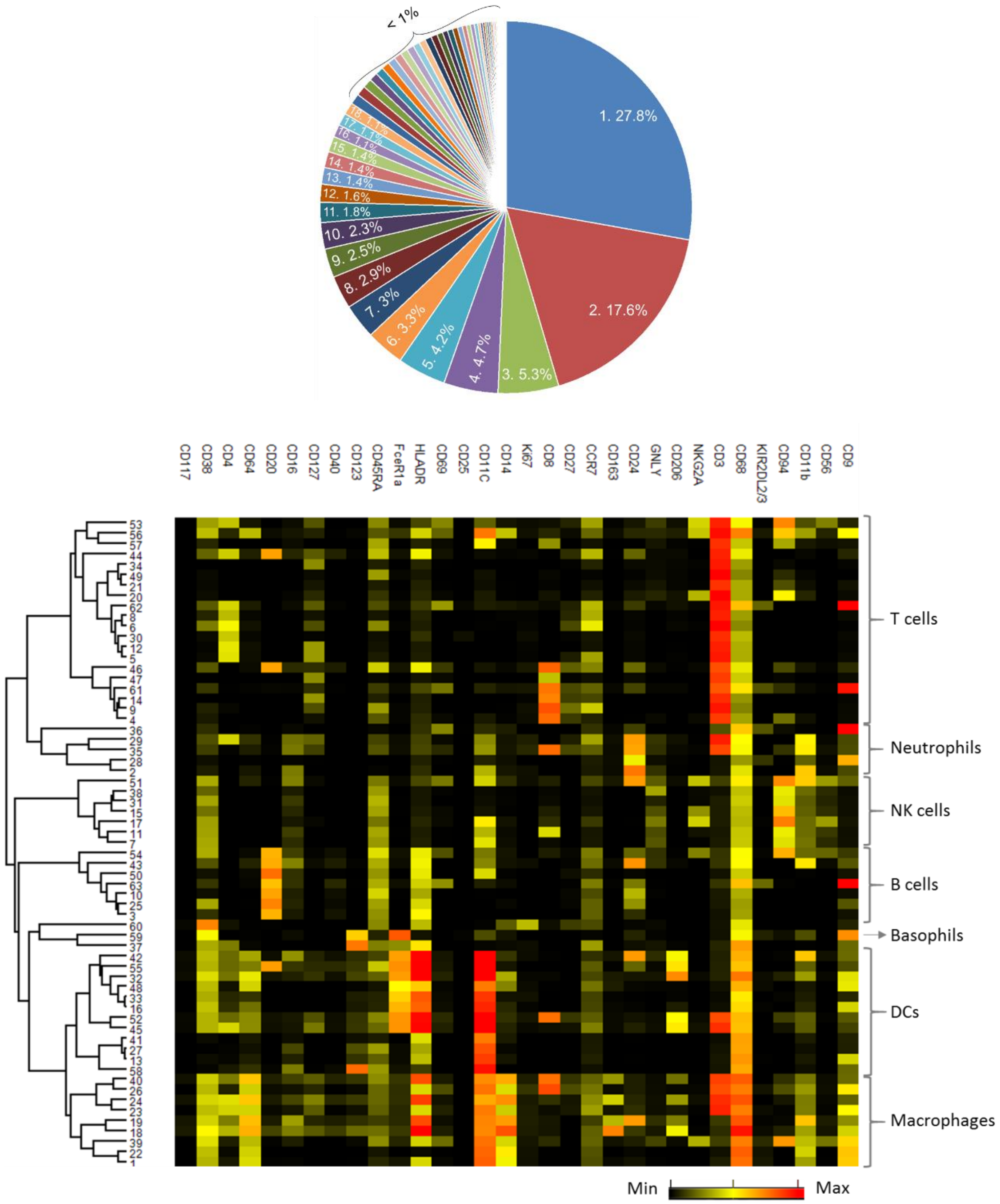

Figure S5. Related to Figure 1. Phenotypic mapping of PBCs. X-shift algorithm identified 63 subpopulations (k = 40) that are ranked in numbers according to their proportions in all PBCs (top). Expression phenotypes and hierarchy of these clusters are shown in heat map (bottom; black represents the minimum, yellow represents median and red represents the maximum expression value).

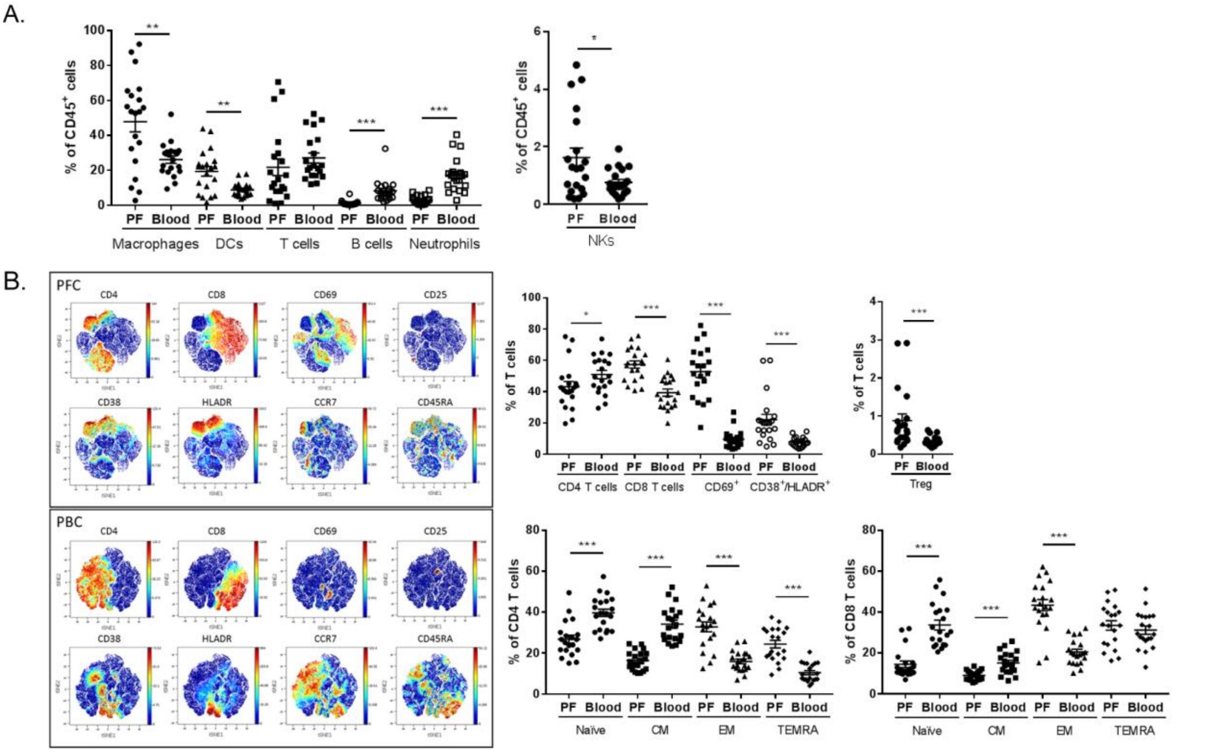

Figure S6. Related to Figure 2. Percentage of major immune cells types in blood and PF samples and expression of functional markers. (A) The frequency of macrophages, DCs, total T cells, B cells, neutrophils and NK cells as a percentage of $CD45^{+}$ cells. (B) ViSNE plots and dot plots of T cells from PFCs and PBCs show composition of cell subsets by expression of markers.

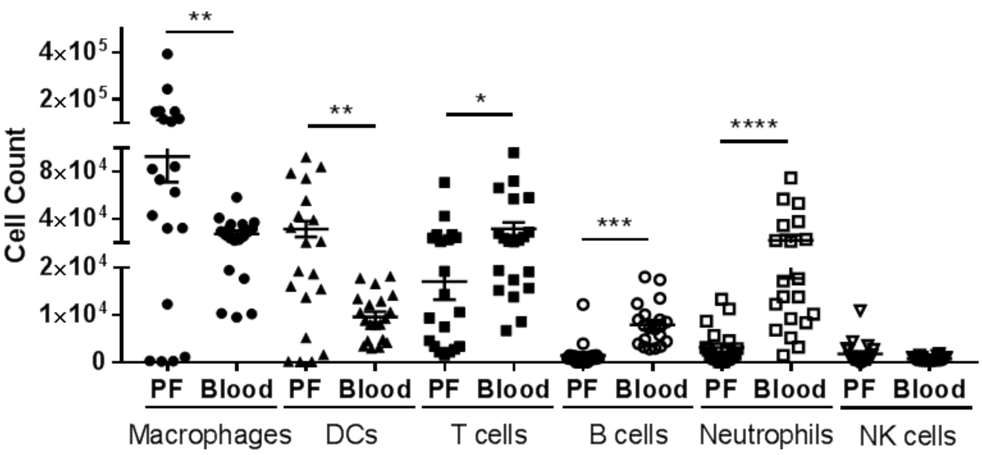

Figure S7. Related to Figure 2. Cell counts show changes of major cell populations in PF compared to peripheral blood. Means ± SEM are shown. Wilcoxon signed-rank test was used in comparison between PF and blood samples. * $p < 0.05$; ** $p < 0.01$; *** $p < 0.001$; **** $p < 0.0001$.

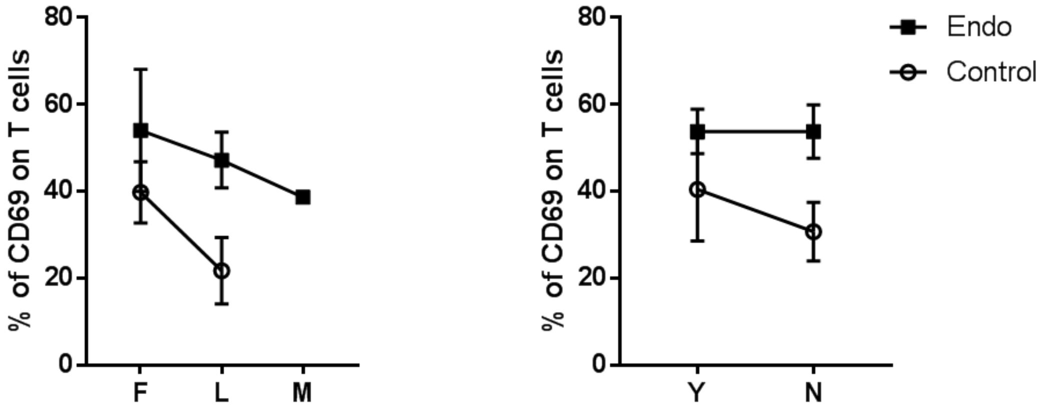

Figure S8. Related to Figure 3. Differential expression of CD69 in endometriosis was not affected by menstruation or hormone. Effects of menstrual cycle (left) and hormone treatment (right) on disease status were evaluated by two-way ANOVA which showed no significant interactions. Means ± SEM are shown in the plots. Menstrual cycle: F, Follicular Phase; L, Luteal Phase; M, Menstruation. Hormone treatment: Y, Yes; N, No. Disease status: Endo, Endometriosis.

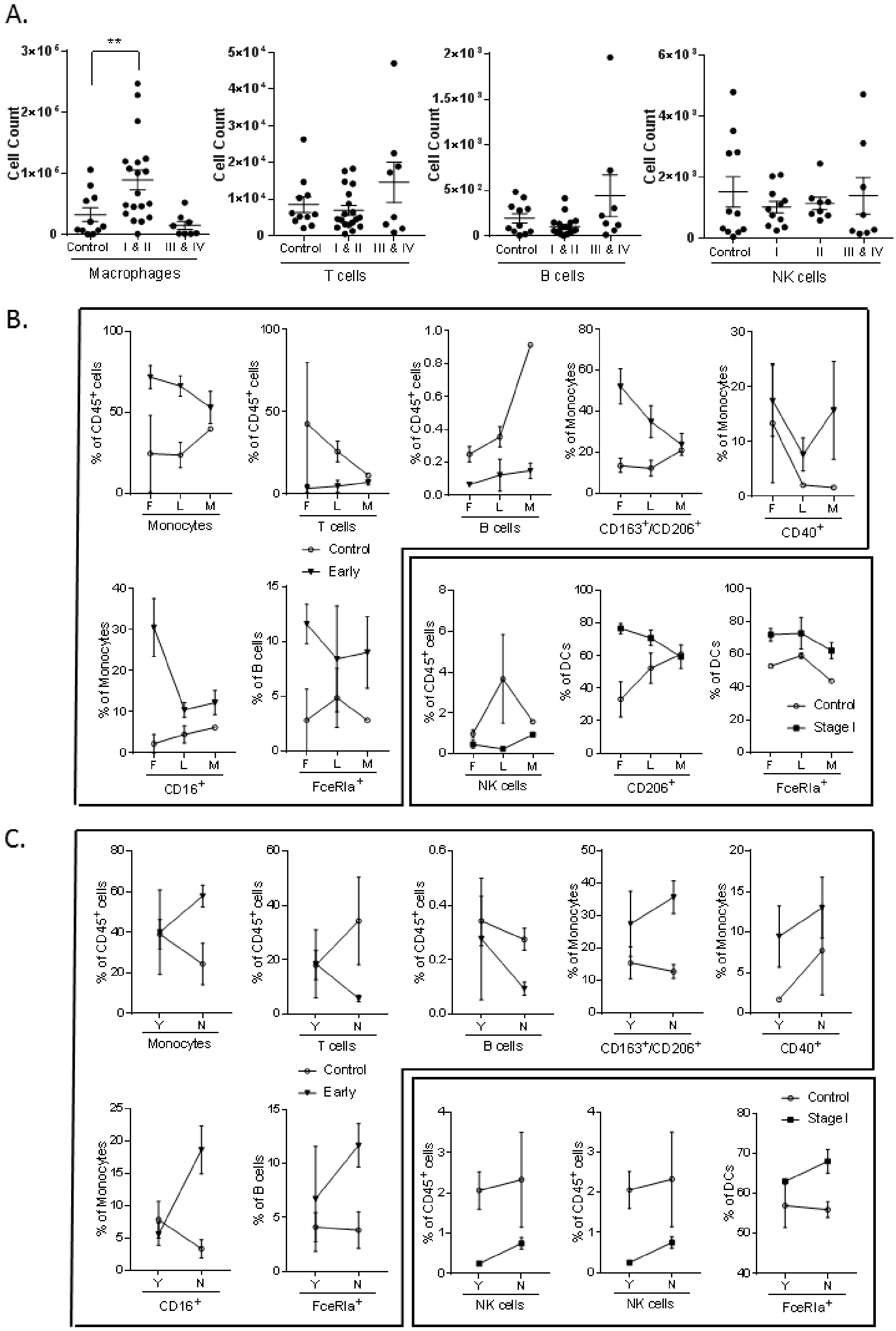

Figure S9. Related to Figure 4. A. Cell counts of major cell subtypes in PFCs at disease stages. Mann Whitney U test was used. ** $p < 0.01$. Effects of menstruation **(B)** and hormone **(C)** on disease status for the significant changes in Figure 4. Menstrual cycle and hormone treatment were evaluated by two-way ANOVA which showed no significant interactions with disease stages. Means ± SEM are shown in the plots. Menstrual cycle: F, Follicular Phase; L, Luteal Phase; M, Menstruation. Hormone treatment: Y, Yes; N, No. Disease status: Endo, Endometriosis.

A.

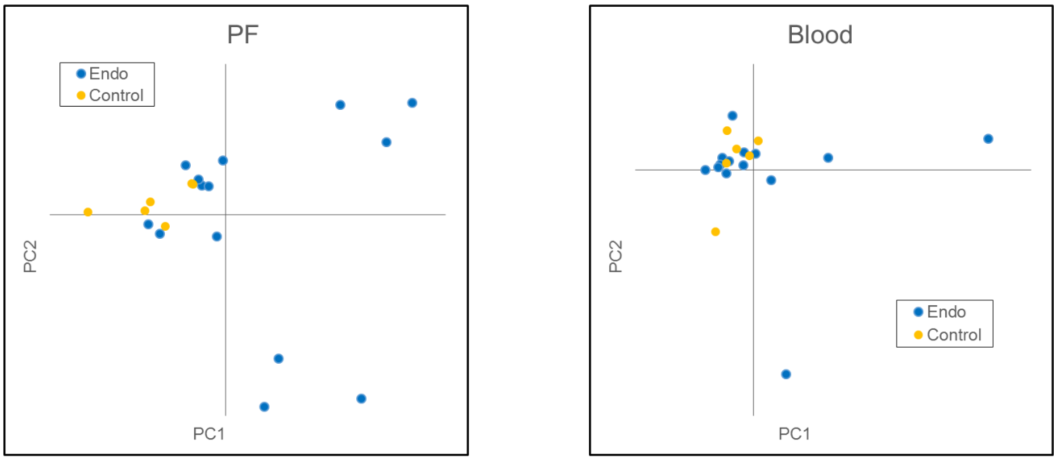

B.

No.	Parameters	Means in Endo	Means in control
1	CD163 on Macrophages	76.00	20.77
2	CD206 on Macrophages	42.84	11.71
3	CD40 on Macrophages	2.28	1.70
4	CD16 on Macrophages	4.75	2.75
5	CD206 on DCs	131.30	57.12
6	FceR1a on DCs	127.20	82.50
7	CD69 on T cells	13.71	4.27
8	CD69 on CD4 T cells	8.51	3.69
9	CD69 on CD8 T cells	15.75	6.87
10	FceR1a on B cells	4.42	2.01

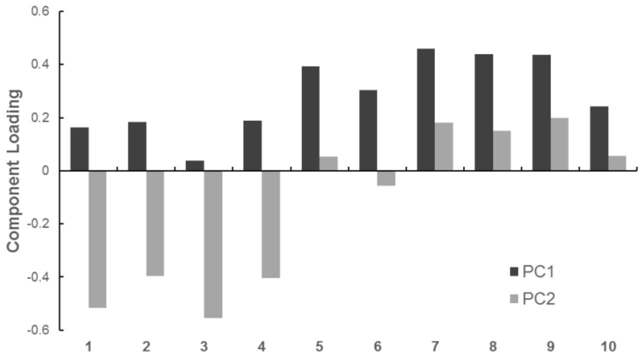

Figure S10. Related to Figure 4. A. PCA separates endometriosis (Endo) and control in PF but not blood samples. Expression values of 10 selected parameters were used for the analysis, results from which are plotted against PC1 and PC2 for PF (left) and blood (right) samples. **B.** The PCA parameter (marker expression values) loadings for the first two components in PF. Expression values of 10 markers were selected for PCA (left). Their loadings to PC1 and PC2 in the analysis were plotted (right), showing that CD69 expression on T cells and M1/M2 activation markers on macrophages are the best discriminators on PC1 and PC2, respectively.

A.

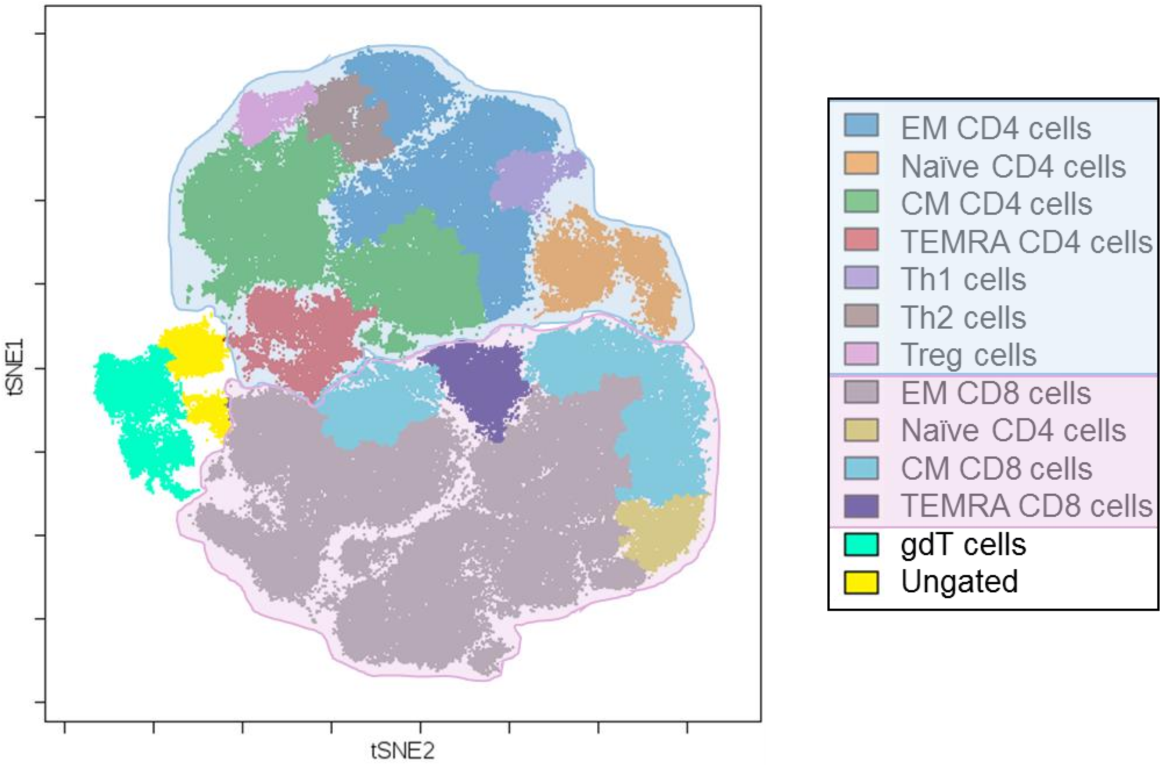

B.

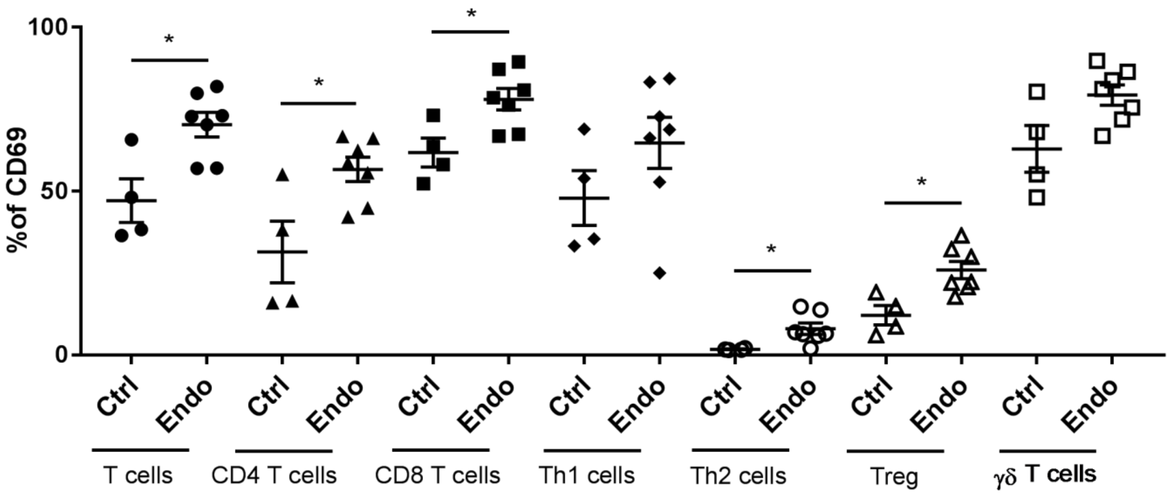

Figure S11. Related to Figure 6. ViSNE plot showing composition of T cells (A) and comparison of CD69 abundance on T cell lineages between control and endometriosis samples from PF (B). ViSNE plot was generated using T cells from all PF samples combined. Means ± SEM are shown in scatter plots. Mann Whitney U test was used. * $p < 0.05$.
