# Supplementary material for: Mass cytometry analysis reveals a distinct immune environment in peritoneal fluid in endometriosis: a characterisation study
Source: BMC Med. 2020 Jan 7;18:3. doi: 10.1186/s12916-019-1470-y (PMC6945609; doi:10.1186/s12916-019-1470-y)
Supplement: Supplementary file 2 — Additional file 2. Related to Fig. 1. Patient-by-patient minimum spanning tree plots showing cell clustering of PF and blood samples. [file 12916_2019_1470_MOESM2_ESM.pdf]

PF sample 1

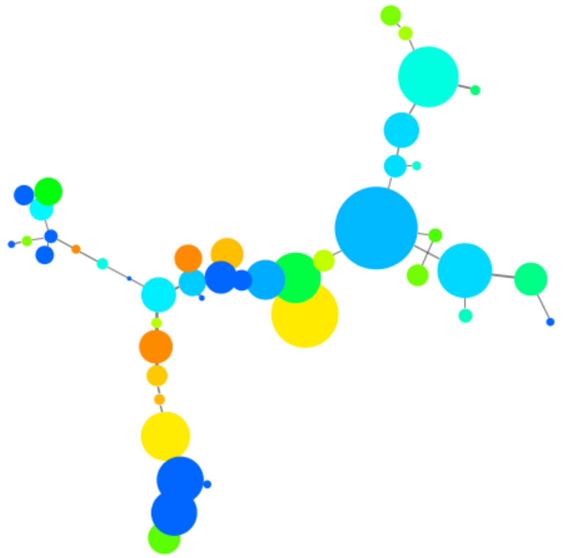

PF sample 2

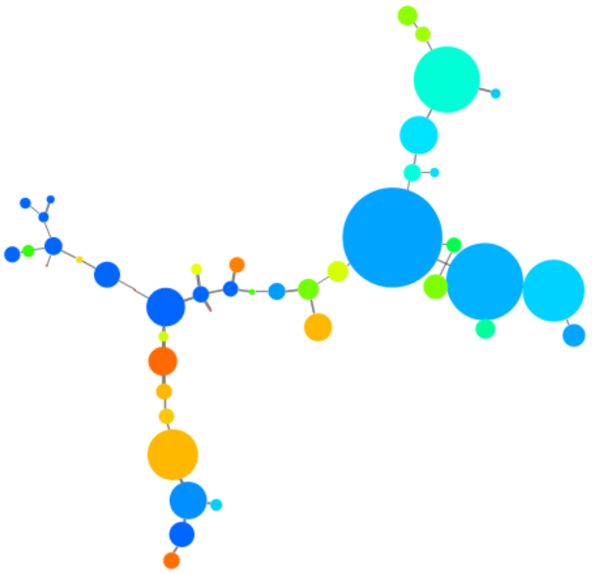

PF sample 3

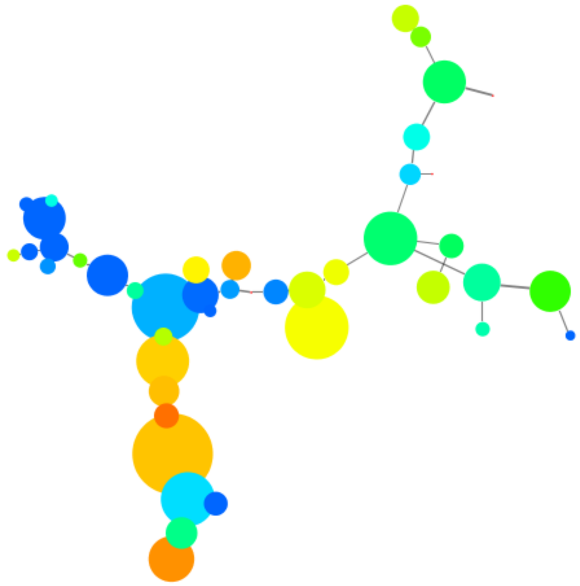

PF sample 4

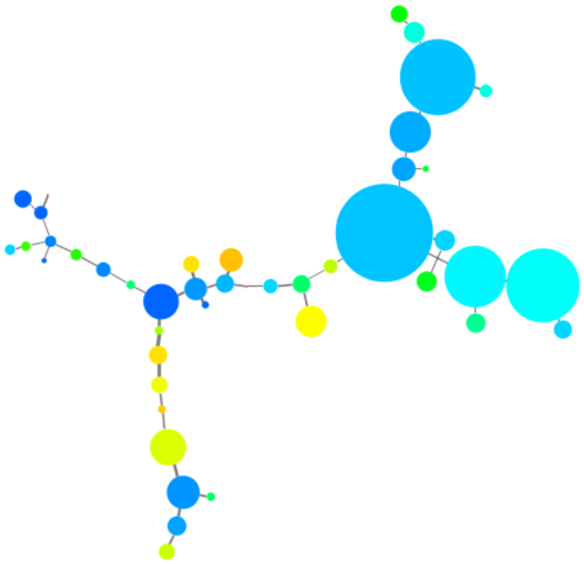

PF sample 5

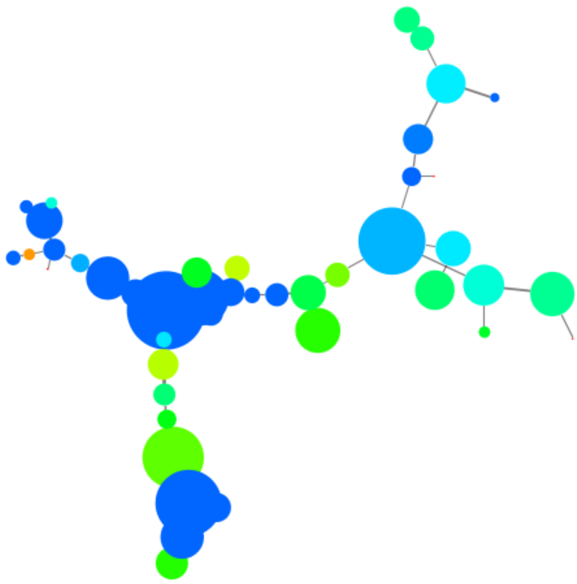

PF sample 6

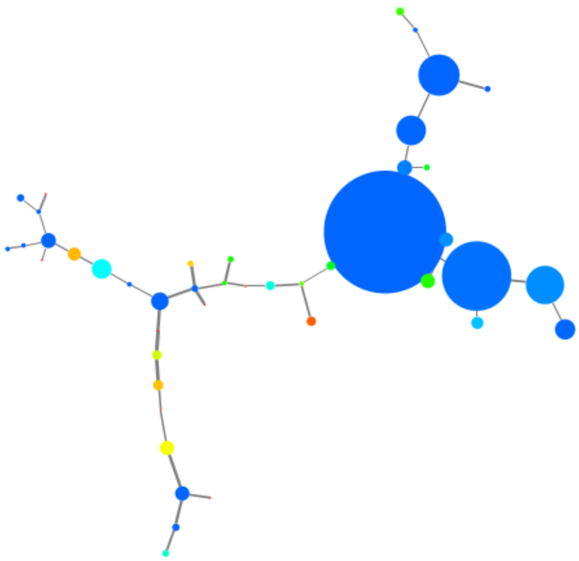

PF sample 7

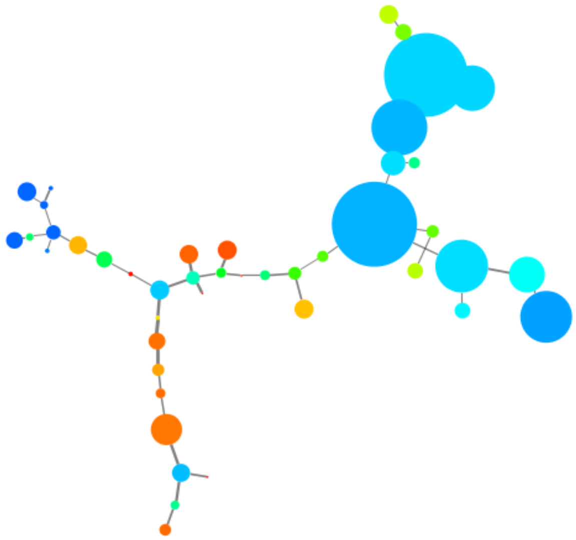

PF sample 8

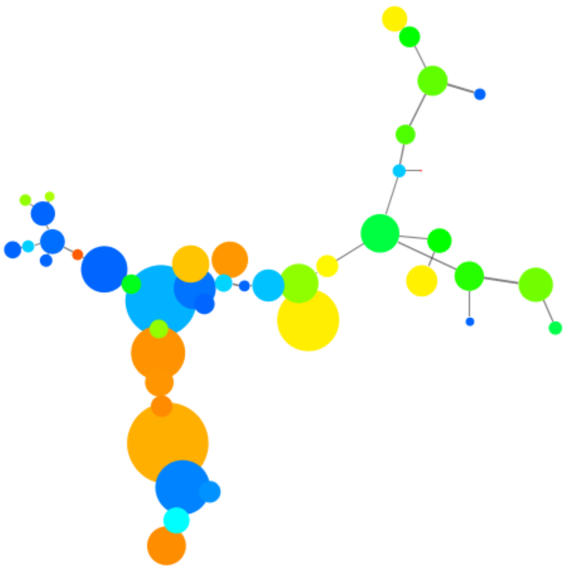

PF sample 9

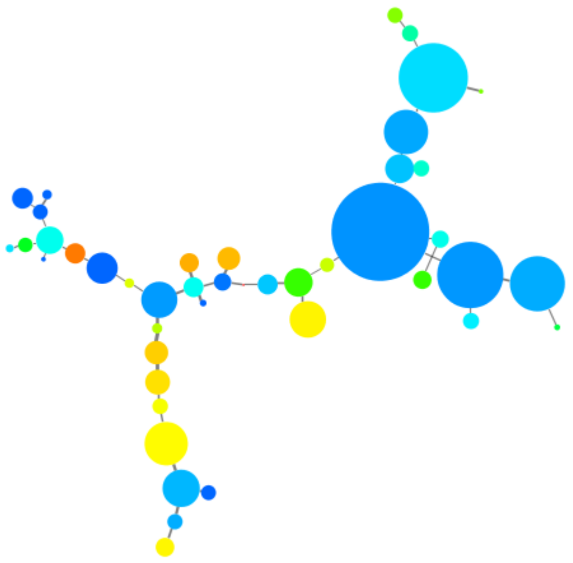

PF sample 10

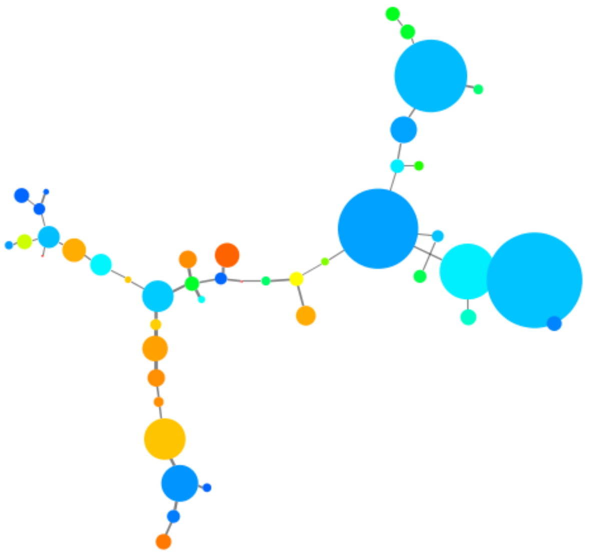

PF sample 11

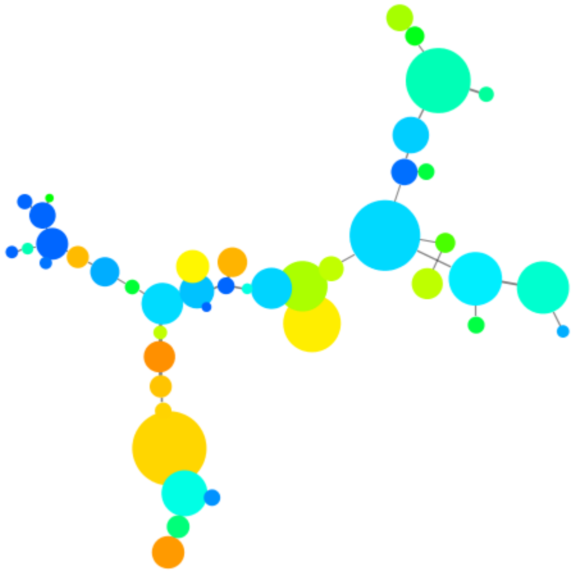

PF sample 12

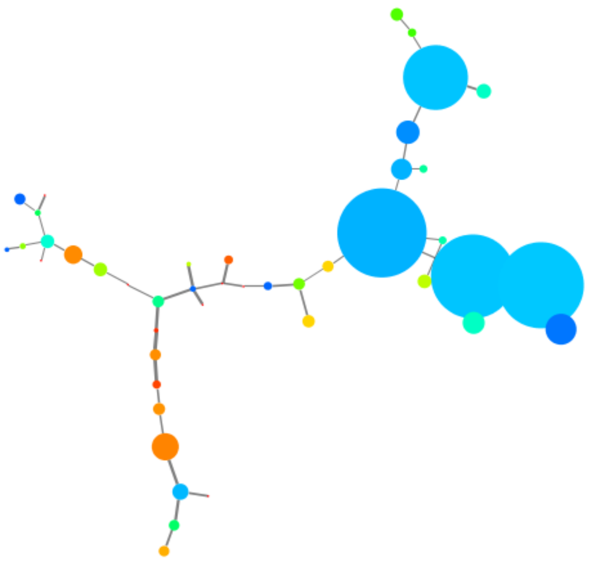

PF sample 13

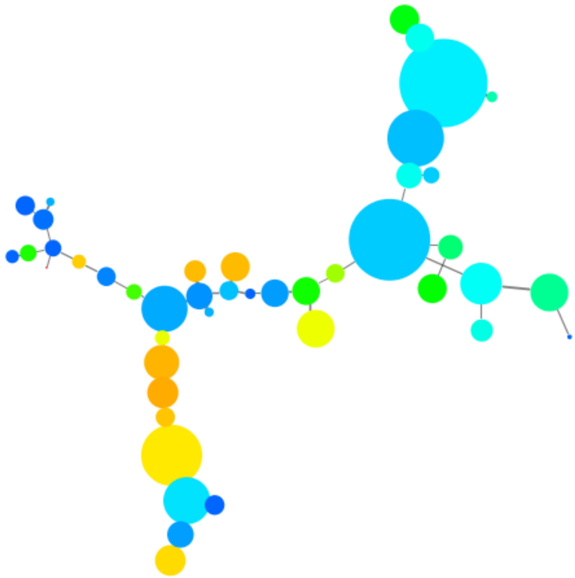

PF sample 14

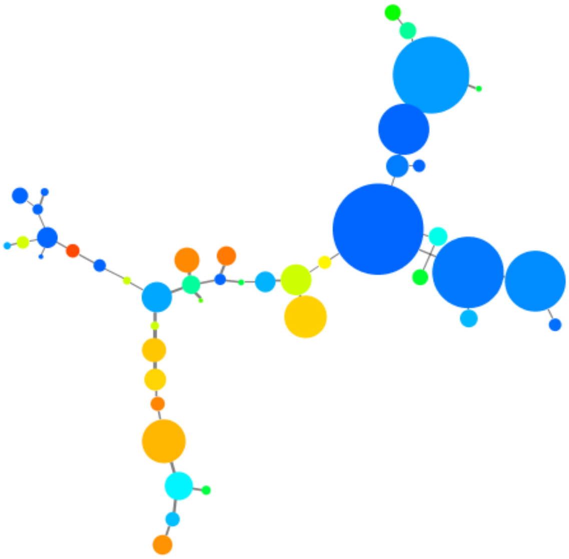

PF sample 15

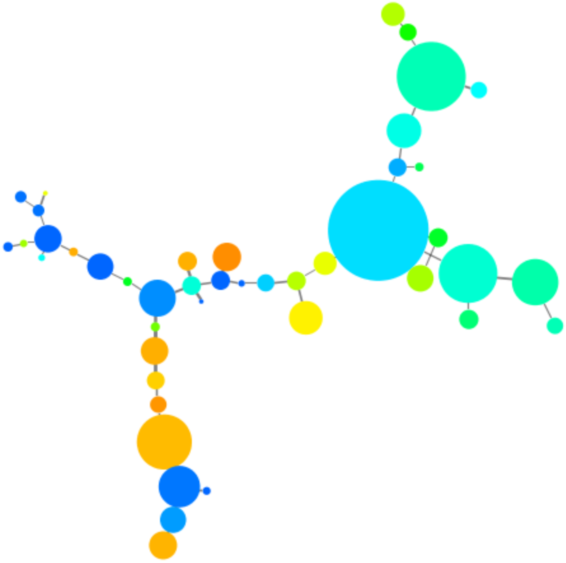

PF sample 16

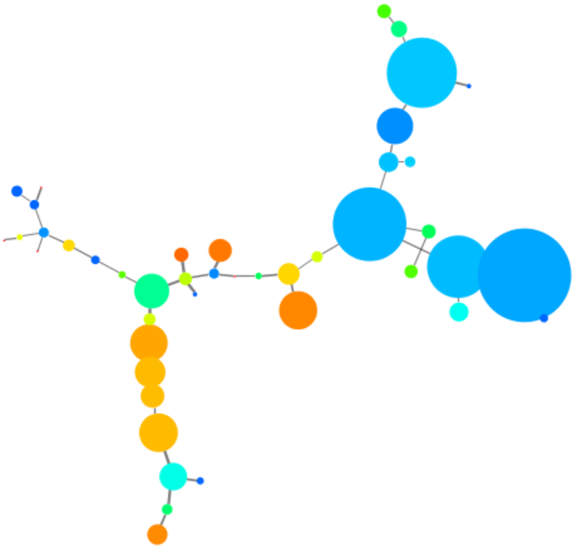

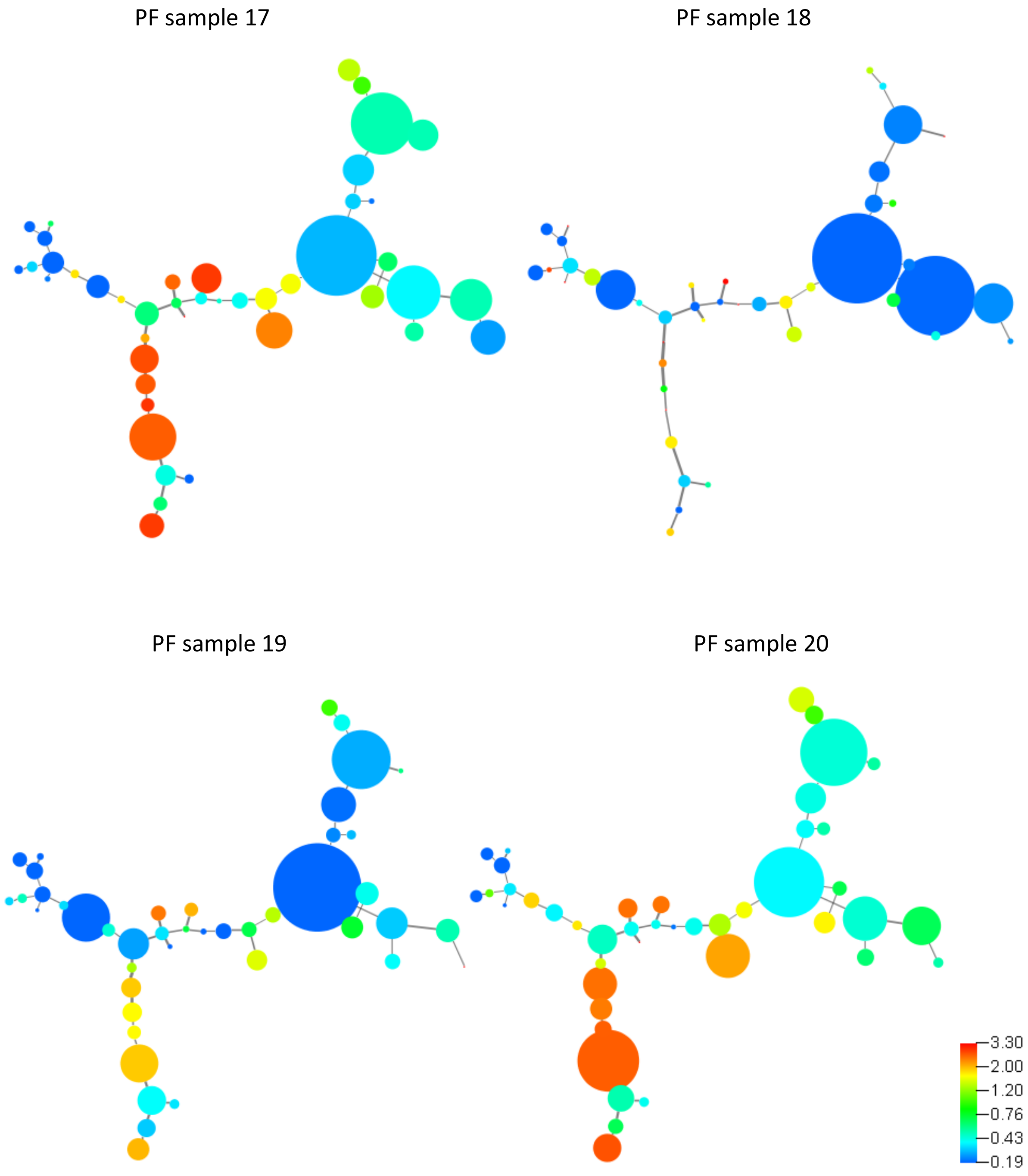

Figure 1. Minimum spanning tree plots showing cell clustering of PF samples. Each node represents a cell cluster and node size indicates abundance of the cluster. Colour scale indicate intensities of CD69.

Blood sample 1

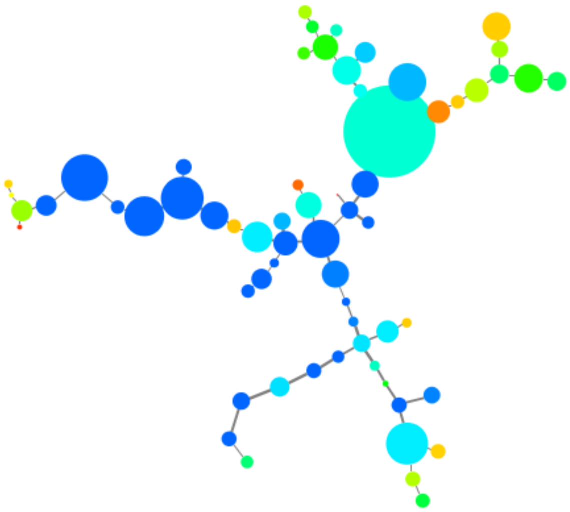

Blood sample 2

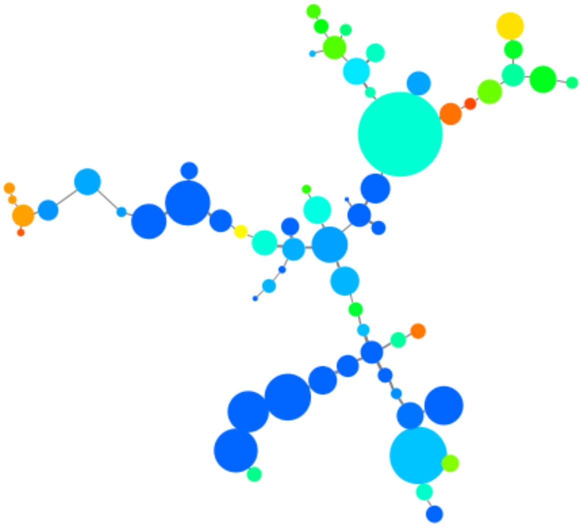

Blood sample 3

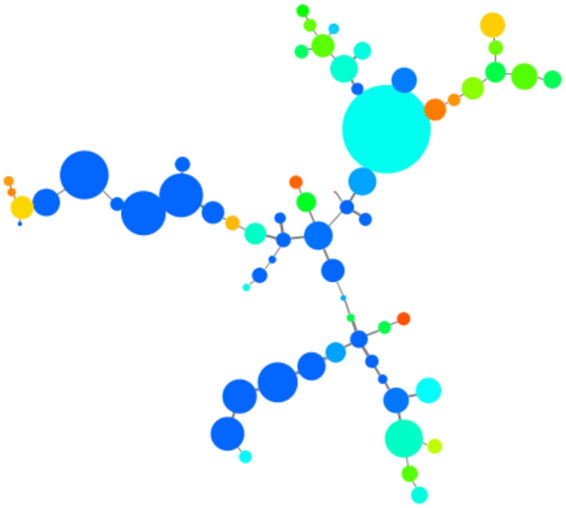

Blood sample 4

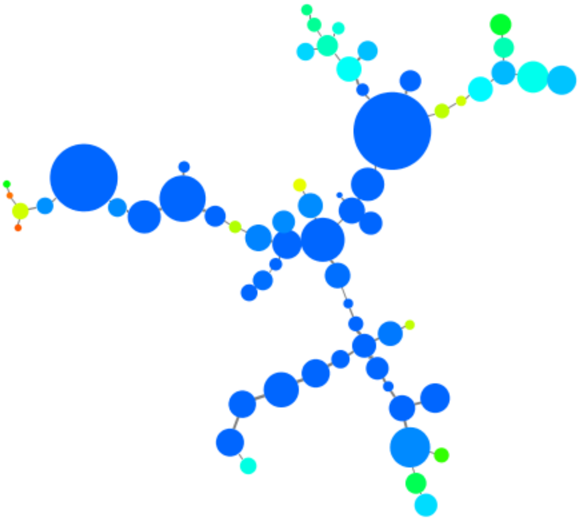

Blood sample 5

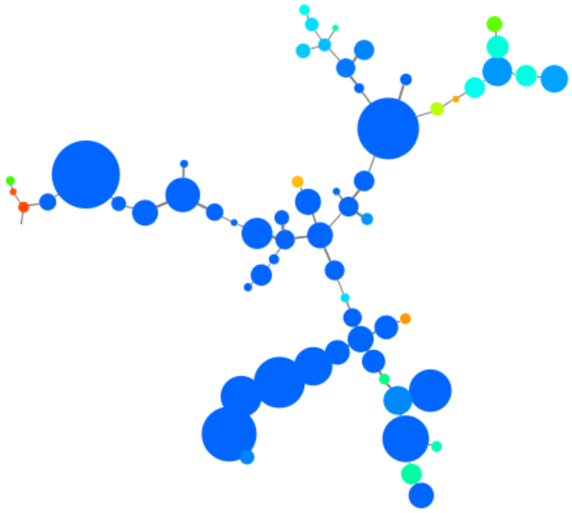

Blood sample 6

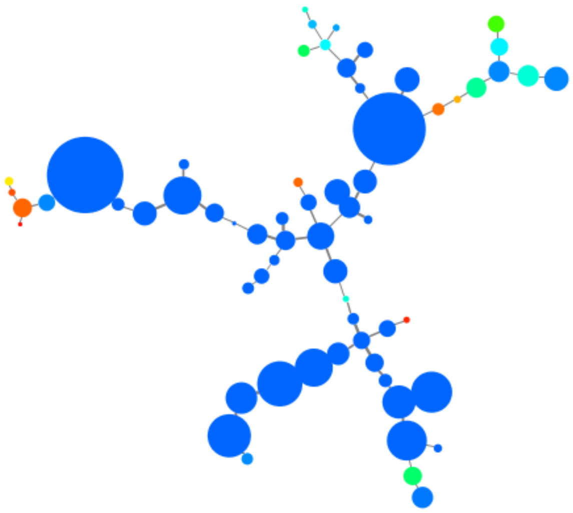

Blood sample 7

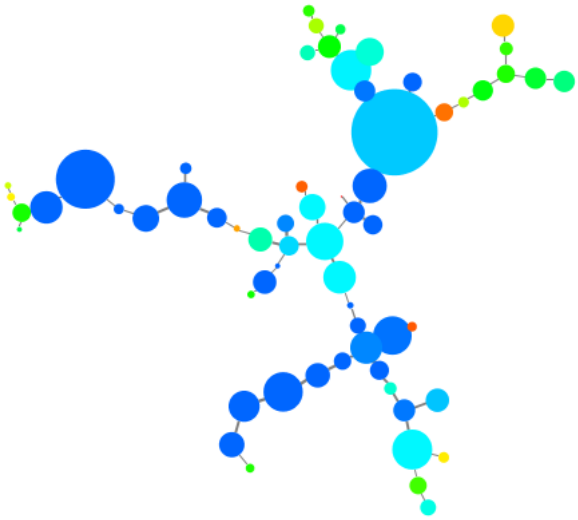

Blood sample 8

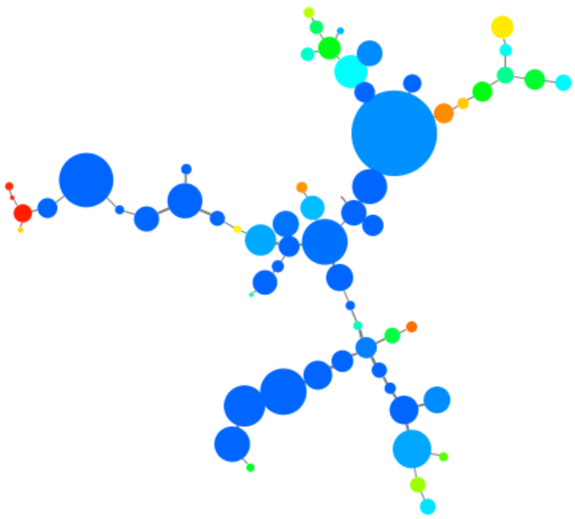

Blood sample 9

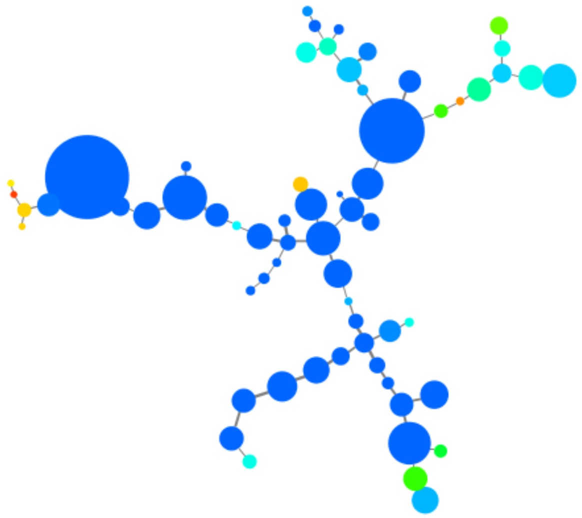

Blood sample 10

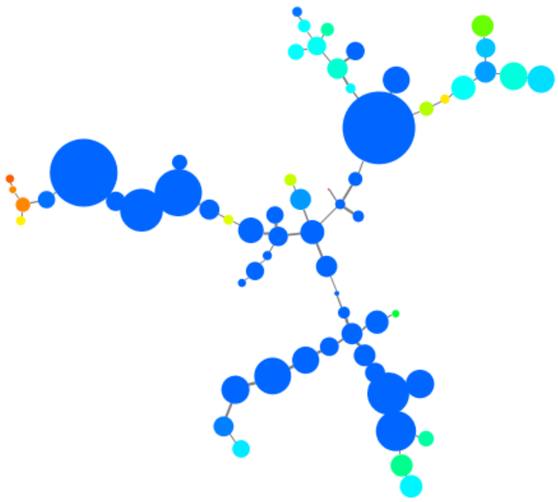

Blood sample 11

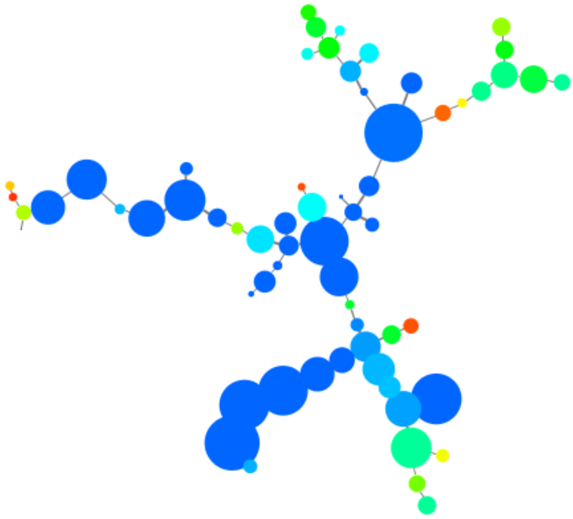

Blood sample 12

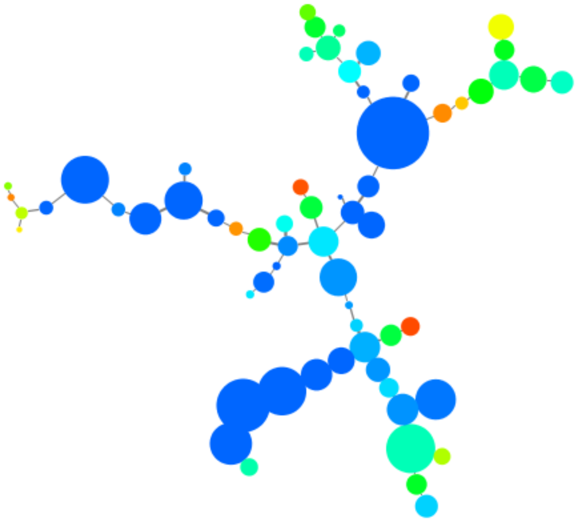

Blood sample 13

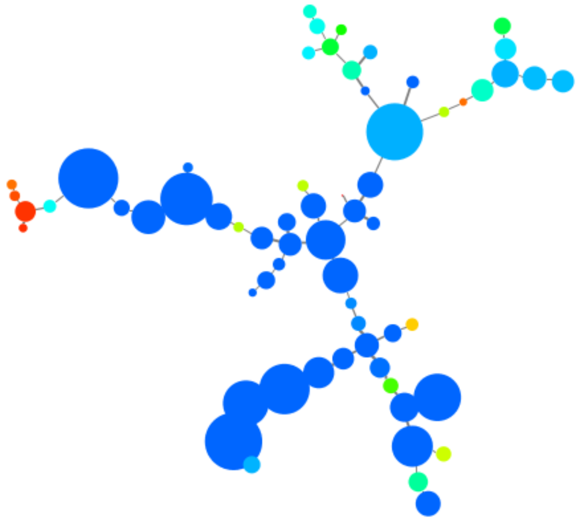

Blood sample 14

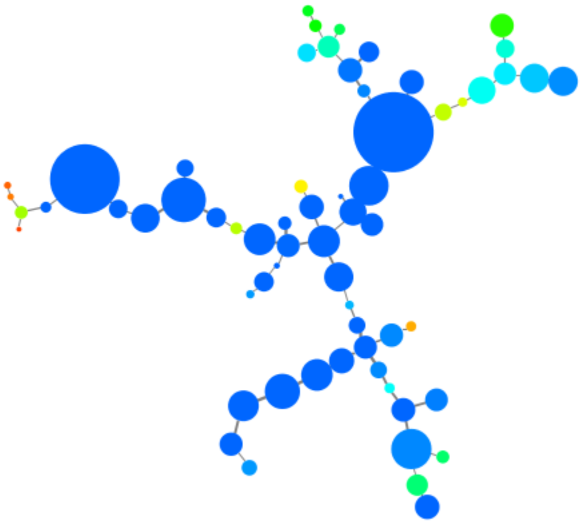

Blood sample 15

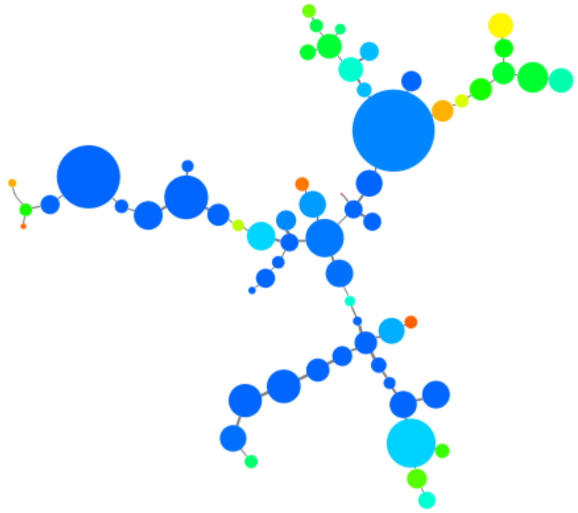

Blood sample 16

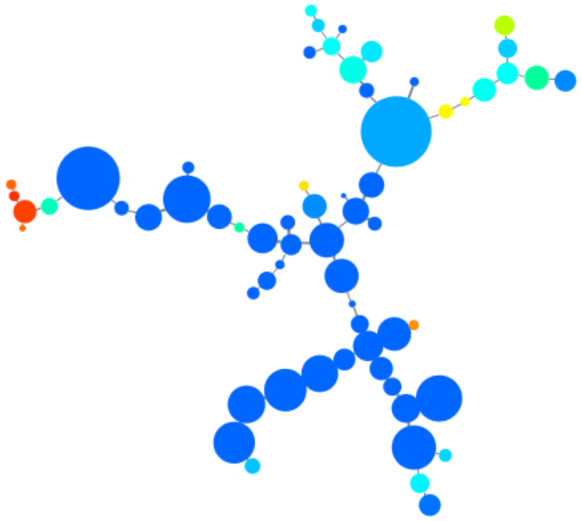

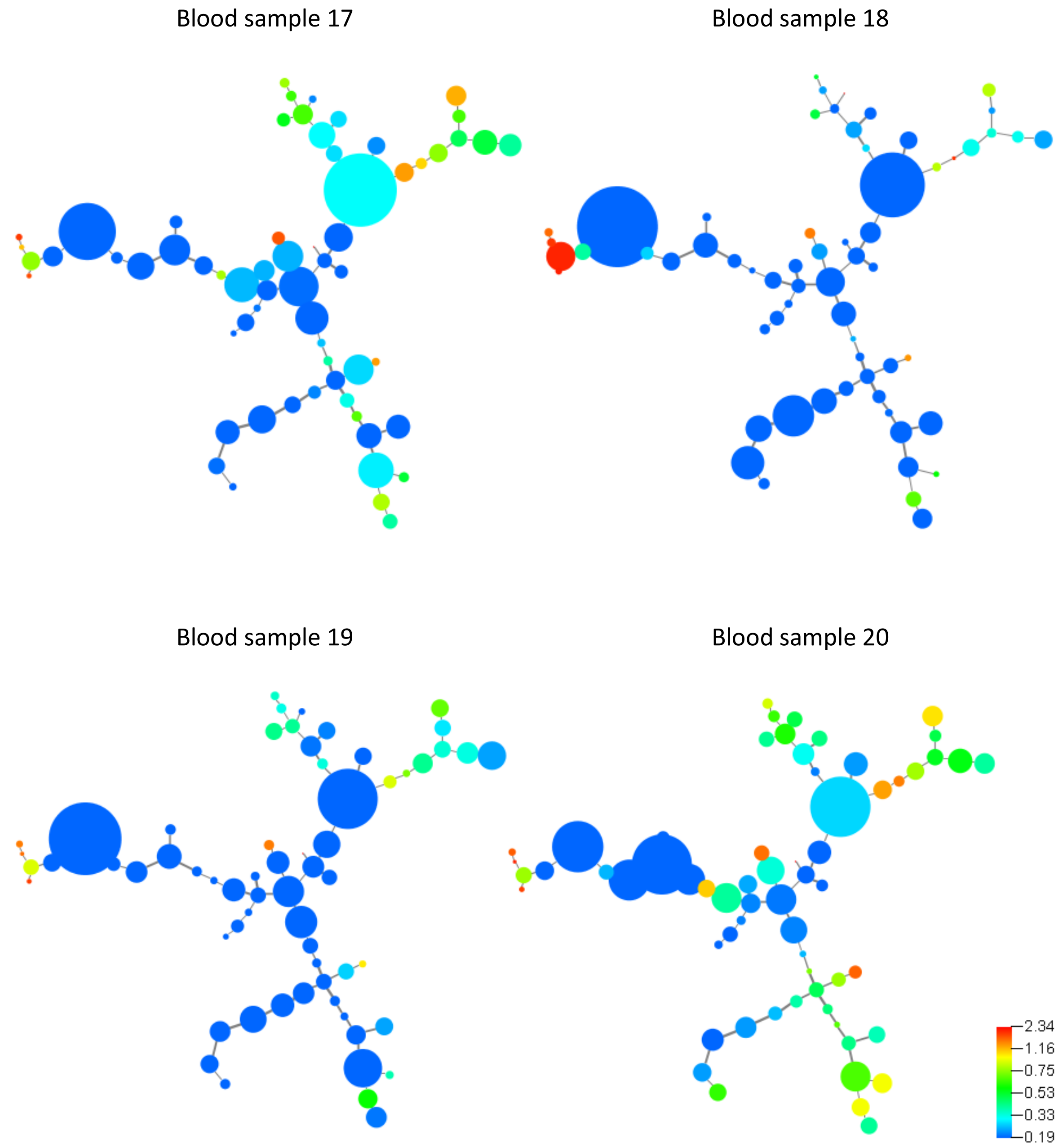

Figure 2. Minimum spanning tree plots showing cell clustering of blood samples. Each node represents a cell cluster and node size indicates abundance of the cluster. Colour scales indicate intensities of CD69.
